# ADT-030, a novel PDE10 inhibitor, demonstrates potent antitumor activity in pancreatic ductal adenocarcinoma

**DOI:** 10.64898/2026.02.11.705411

**Published:** 2026-02-13

**Authors:** Dhana Sekhar Reddy Bandi, Ganji Purnachandra Nagaraju, Sujith Sarvesh, Jeremy B. Foote, Adam B. Keeton, Xi Chen, Veronica Ramirez-Alcantara, Thomas Holmes, Mehmet Akce, Anju Singh, Craig M. Powell, Santosh Behera, Asfar S. Azmi, Elmar Nurmemmedov, Ivan Babic, Gregory S. Gorman, Lori Coward, Donald J. Buchsbaum, Yulia Y. Maxuitenko, Gary A. Piazza, Bassel F. El-Rayes

**Affiliations:** 1 Division of Hematology and Oncology, University of Alabama at Birmingham, Birmingham, AL 35233, USA.; 2 Department of Microbiology, University of Alabama at Birmingham, Birmingham, AL 35294, USA.; 3 Drug Discovery and Development Department, Harrison College of Pharmacy, Auburn University, Auburn, AL 36849, USA.; 4 Health Biobank and Histology Core Facility, Mitchell Cancer Institute, University of South Alabama, Mobile, AL 36604, USA.; 5 Department of Neurobiology, University of Alabama at Birmingham, Birmingham, AL 35294, USA.; 6 Department of Biotechnology, National Institute of Pharmaceutical Education and Research, (NIPER), Ahmedabad, Gujarat- 382355, India.; 7 Department of Oncology, Karmanos Cancer Institute, Wayne State University School of Medicine, Detroit, MI 48201, USA.; 8 CellarisBio LLC, San Diego, CA 92121, USA; 9 Department of Pharmaceutical, Social and Administrative Sciences, McWhorter School of Pharmacy, Samford University, Birmingham, AL 35229, USA.; 10 Department of Obstetrics and Gynecology, University of Alabama at Birmingham, Birmingham, AL 35233, USA.; 11 ADT Pharmaceuticals, LLC, Orange Beach, AL 31691, USA.

**Keywords:** KRAS-mutant PDAC, targeted therapy, immune checkpoint inhibition, T and NK cell activation, PKA/PKG signaling, myeloid polarization, PDE10, β-catenin

## Abstract

Phosphodiesterase 10 (PDE10) was previously reported to be overexpressed in various cancers and essential for cancer cell proliferation and survival. Here, we studied a novel PDE10 inhibitor, ADT-030, and found it to potently and selectively inhibit KRAS mutant PDAC cell proliferation and clonogenicity by inducing G2/M arrest and apoptosis. ADT-030 also inhibited motility of PDAC cells *in vitro*. These effects were mediated by increased cAMP/cGMP levels and activation of PKA/PKG. The growth inhibitory activity of ADT-030 was associated with reduced β-catenin and RAS signaling. Notably, ADT-030 also inhibited the growth of KRAS^G12D^ and KRAS^G12C^ mutant PDAC cells resistant to allele-specific KRAS inhibitors. Oral administration of ADT-030 significantly suppressed tumor growth, reduced lung and liver metastasis, and increased survival without systemic toxicity in syngeneic and patient-derived xenograft (PDX) PDAC models. ADT-030 also increased chemotherapy response in orthotopic PDAC models. Immune phenotyping and single-cell RNA sequencing revealed remodeling of the tumor microenvironment by ADT-030 with a more favorable immune suppressive profile to activate anti-tumor immunity. These results show that ADT-030 is a promising drug development candidate for the treatment of KRAS-mutant PDAC capable of simultaneously targeting key oncogenic signaling pathways, resulting in tumor-intrinsic and immunomodulatory effects.

## Introduction

Pancreatic ductal adenocarcinoma (PDAC) is the fourth most common cause of cancer-related mortality in the U.S., with a five-year survival of under 13% ^1^. Survival of patients with metastatic PDAC remains poor even with the current chemotherapeutic regimens such as FOLFIRINOX or gemcitabine and nab-paclitaxel ^2^. The aggressive nature of PDAC is mainly attributed to activation of multiple compensatory signaling pathways driving proliferation and survival, along with a hypoxic microenviroment driven by dense desmoplastic stroma and decreased vascular perfusion ^3–7^. Although allele-specific KRAS inhibitors have demonstrated promising activity in early-phase clinical trials in patients with PDAC, the development of resistance remains a major challenge and highlights the need to identify new therapeutic targets and agents with broader activity ^8–10^. A better understanding of the complexity of oncogenic signaling, the importance of stroma, and the role of immune evasion in PDAC progression is critical for the development of more effective target-directed drugs for the treatment of PDAC ^11^.

Mutations in the KRAS gene have been reported in about 90% of PDAC patients, with the majority at the 12^th^ codon (KRAS^G12D^, KRAS^G12V^, and KRAS^G12C^) ^12^. These mutations result in the constitutive activation of downstream pathways such as RAS/RAF/MEK and PI3K/AKT/mTOR signaling to promote the proliferation, survival, and metabolic reprogramming of PDAC ^13^. Although the mutation frequency of *CTNNB1* is relatively low in PDAC, β-catenin signaling is aberrantly activated from WNT overexpression, which, along with KRAS mutations, contributes to the aggressive behavior of PDAC ^6^. KRAS has been reported to form a complex with β-catenin to modulate the phosphorylation of the transcription factor TCF4, leading to crosstalk between these two oncogenic signaling pathways ^14,15^. In addition, both β-catenin and RAS signaling have been reported to be activated with gemcitabine treatment, suggesting that these pathways play a major role in therapy resistance in PDAC ^16^. Given the interactions between RAS and β-catenin in PDAC, a strategy that targets both pathways with single inhibitor could offer more robust therapeutic approach. Emerging evidence also suggests that simultaneous inhibition of multiple oncogenic pathways not only increases the potential for efficacy of target-directed anticancer drugs but also reduces the potential for resistance by overcoming compensatory signaling mechanisms ^17,18^. KRAS mutated tumors can utilize β-catenin signaling to maintain a stem-like, immune-depleted, niche within the tumor immune microenvironment (TiME) resulting in relapse following chemotherapy. Targeting both pathways with one inhibitor could also sensitize cancer cells to undergo apoptosis while modulating the TiME to favor immune activation leading to inhibition of tumor growth ^19^.

Phosphodiesterase (PDE) isoenzymes hydrolyze and inactivate the second messengers, cyclic adenosine monophosphate (cAMP) and cyclic guanosine monophosphate (cGMP) ^20^. Although understudied, PDEs have been reported to play a role in the initiation and progression of PDAC and other cancers ^21,22^. Notably, the cAMP/PKA and cGMP/PKG signaling axes have been reported to suppress MAPK signaling downstream from KRAS ^23,24^. In addition, PDE isoenzymes have been shown to regulate β-catenin signaling, which can also influence RAS signaling ^25–27^. Several PDE isozyme families, most notably, PDE4, PDE5, and PDE10 have been investigated as anti-cancer targets inhibition of which can impact cancer cell proliferation, survival, and immune responses ^22,28^. Notably, isozyme-specific inhibitors of the dual cAMP/cGMP degrading PDE10 isozyme and gene silencing approaches have been reported to selectively inhibit the proliferation and induce apoptosis of cells from colon, lung, and ovarian cancers through activating cGMP/PKG signaling and disrupting RAS signaling and WNT/β-catenin-mediated transcription ^25,27,29,30^. These findings established the basis for our hypothesis that ADT-030, a novel PDE10 inhibitor with properties distinct from known PDE10 inhibitors developed for CNS disorders, can block both RAS and β-catenin signaling and result in tumor inhibition and modulation of the TiME in PDAC.

## Materials and methods

### ADT-030 synthesis

The synthesis of ADT-030 [(S,Z)-2-(5-methoxy-2-methyl-1-(3,4,5-trimethoxybenzylidene)-1H-inden-3-yl)-N-(1-methylpyrrolidin-3-yl)acetamide] is based on a procedure originally described in US patent 20200223815 using 3-(4-methoxyphenyl)-2-methylacrylic acid as the starting material.

### Human scRNA-seq datamining

The Single Cell RNA seq Pancreatic Cancer Atlas R Data Serialization (RDS) file (https://zenodo.org/records/14199536) was downloaded ^31^. This dataset has normalized and scaled scRNA-seq (10x genomics sequencing) data from 12 studies containing 229 patients across the groups. The ductal cells were identified from the main dataset and the expression levels of PDE10 was queried across the tissue samples (donor, adjacent normal, primary tumor, and metastatic lesion) as described in the previously published paper^31^.

### Cellular target engagement assay

HEK293 cells expressing PDE10 fused to the MICRO-TAG reporter were subjected to a temperature-series denaturation assay to determine the aggregation midpoint under cellular conditions as previously described ^32^. Cells were heated for 10 min across a defined temperature range, followed by non-denaturing lysis and fluorescence complementation quantification. Fitting the resulting thermal curve yielded a Tagg_50_ of 44°C for PDE10. This defined Tagg_50_ provided the fixed challenge temperature for subsequent experiments to determine if ADT-030 binds PDE10 in intact cells.

### Cell culture

Panc-1 (ATCC# CRL-1469; RRID:CVCL_0Q68), AsPC-1 (ATCC# CRL-1682; RRID:CVCL_0152), Panc 02.03 (ATCC# CRL-2553; RRID:CVCL_1633), Panc 10.05 (ATCC# CRL-2547; RRID:CVCL_1639), MIA PaCa-2 (ATCC# CRL-1420; RRID:CVCL_0428), BxPC-3 (ATCC# CRL-1687; RRID:CVCL_0186), and KLE (ATCC# CRL-1622; RRID:CVCL_1329) cell lines were obtained from American Type Culture Collection (ATCC, Manassas, VA, USA) and maintained as recommended. Mouse PDAC cell line 2838c3 (Kerafast# EUP013-FP; RRID:CVCL_YM18) was purchased from Kerafast (Boston, MA, USA). MKN1 (Accegen# ABC-TC0685; RRID:CVCL_1415) cell line was purchased from Accegen Inc (Fairfield, NJ, USA). Dr. Gregory Lesinski, Emory University, USA, gifted the KPC cell line. Dr. Denis C Guttridge, Medical University of South Carolina, USA, gifted the KPCML1 cell line. All cells were grown in appropriate medium as recommended by the ATCC and Kerafast with either Dulbecco’s Modified Eagle Medium (DMEM; ATCC# 30–2002) or Roswell Park Memorial Institute (RPMI)-1600 Medium (RPMI; ATCC# 30–2001) supplemented with 10% fetal bovine serum (FBS; ATCC# 30–2020) and 1% penicillin/streptomycin (ATCC# 30–2300) under 5% CO_2_. Additionally, MIA PaCa-2 cells received 2.5% horse serum (Thermo# 26050088).

### Phosphodiesterase assay

The enzymatic activity of recombinant PDE10 was measured using the Immobilized Metal Affinity Particle (IMAPTM) fluorescence polarization (FP) progressive binding system (Molecular Devices; San Jose, CA; USA) as previously described to determine the inhibitory effect of ADT-030 ^33^. FP was measured using a Synergy H4 Hybrid plate reader (BioTek; Santa Clara, CA; USA). Recombinant PDE10 was purchased from BPS Biosciences (San Diego, CA; USA).

### Proliferation assay

Human and murine PDAC cells with KRAS mutations (KRAS^G12D^ and KRAS^G12C^) and wild-type cells (BxPC-3) were plated at a density of 5×10^3^ cells/well in 96-well plates. After 20 hrs, cells were treated with ADT-030 or PF-2545920 (a known PDE10 inhibitor) ^34^ in a dose-dependent manner. After 72 hrs, the medium was removed, 10-μL methylthiazole tetrazolium (MTT; 5 mg/mL in PBS; Sigma-Aldrich# 475989) was added, and cells were incubated for another 2 hrs at 37 °C. The resulting formazan crystals were solubilized in 100 μL DMSO (Sigma-Aldrich# D2438), and absorbance was measured at 570 nm with a reference wavelength of 630 nm.

### Clonogenic assay

Mouse derived PDAC cell line 2838c3 (KRAS^G12D^) and human derived cell line MIA PaCa-2 (KRAS^G12C^) were plated in 6-well culture plates (2 × 10^3^ cells/well). The cells were then treated with either DMSO, ADT-030 at 0.5, 1, 2, and 5 μM, or PF-2545920 at 5, 10, 25, 50, and 100 μM every 3 days. After 10 days, the cells were stained with a 0.005% Coomassie Brilliant Blue R-250 solution, and plates were imaged using an Epson Perfection V850 Pro Photo Scanner (USA). The resulting colonies were counted using ImageJ (RRID:SCR_003070).

### Motility assay

To measure effects on cell motility, 2838c3 and MIA PaCa-2 cells were grown in 6-well plates until they reached confluence. The cells were treated with DMSO or ADT-030 (0.5, 1, 2, and 5 μM). A scratch was created using a sterile 10-μL pipette tip, and cell migration was monitored daily using light microscopy. Quantification of cell movement was performed using ImageJ software.

### Apoptosis assay

The binding of annexin V to cells was measured using the PE-Annexin V Apoptosis Detection Kit I (BD Biosciences# 559763), according to the manufacturer’s protocol. Briefly, 2838c3 and MIA PaCa-2 cells were treated with either DMSO or ADT-030 (at 2 and 5 μM) for 72 hrs. After treatment, cells were collected, washed twice with cold 1x PBS (ATCC# 30–2200), and suspended in 1x Binding Buffer. The cells were then stained with 300 μL PE Annexin V FITC and 5-μL of propidium iodide (PI) and incubated for 15 min in the dark. Flow cytometry analysis was performed using a BD LSR Fortessa Flow Cytometer and data were analyzed using FlowJo (RRID:SCR_008520).

### Cell cycle assay

2838c3 and MIA PaCa-2 cells were treated with either DMSO or ADT-030 (2 and 5μM) for 24 hrs and the cells were trypsinized and centrifuged at 1,000x g for 3 min at 4 °C. Cells were then washed with PBS and fixed using 70% ethanol at 4 °C overnight. The following day, the cells were incubated with 1 mL of RNAse solution for 30 min in the dark and stained with PI for 30 min. The cells were then analyzed for cell cycle arrest using a BD LSR Fortessa Flow Cytometer. The experiment was repeated thrice independently, and the results were analyzed using FlowJo.

### Immunoblotting

Whole-cell protein extracts were prepared using RIPA Lysis Buffer (Pierce Chemical, Rockford, IL, USA) containing protease inhibitor cocktail (Roche, Basel, Switzerland) and phosphatase inhibitor cocktail (Sigma-Aldrich, St. Louis, MO, USA). Lysed samples were centrifuged at 12,000 rpm for 40 min, and clarified supernatants were stored at −80 °C. Protein concentrations were determined using the Pierce Bicinchoninic Acid (BCA) protein assay kit. Equal amounts of protein samples were electrophoresed on 4–20% sodium dodecyl sulfate (SDS)- polyacrylamide gels (BIO-RAD, #4568096) and transferred onto PVDF membranes (Invitrogen, #IB34001). The membranes were then incubated with antibodies diluted in 2% Bovine Serum Albumin (BSA, Fisher Scientific, #BP1600) for 2 hrs at room temperature. Primary antibodies were pERK, ERK, pAKT, AKT, pmTOR, mTOR, pP70s6 kinase, p70s6 kinase, pCREB, CREB, Bcl-2, VEGFA, PDE3B, PDE4C, PDE4D, LC3A/B, cleaved PARP, cleaved caspase 3, non phospho β-catenin, β-catenin, pVASP, VASP, and anti-β-actin. Incubation with HRP-linked secondary antibodies (CST, #7074/7076;) at a dilution of 1:3000 in a 2% BSA solution was carried out for 1 hr at room temperature. The signal was then detected on a LI-COR Odyssey DLx Imager using the ECL chemiluminescence detection system (Thermo Fisher Scientific, #34577).

### Measurement of intracellular cAMP and cGMP levels

2838c3 and MIA PaCa-2 cells were treated with ADT-030 at varying doses, harvested, and the intracellular cAMP and cGMP levels were measured. Enzyme immunoassay kits were used to detect cAMP (Cat# 581001, Cayman) and cGMP (Cat# 581021, Cayman) by following manufacturer’s instructions. The results were expressed as picomoles/μg of total protein.

### Immunohistochemistry (IHC) and immunofluorescence (IF)

Paraffin-embedded tumor tissue slides from 2838c3 and KPC orthotopic studies were used for detecting the expression patterns of extracellular matrix (ECM) remodeling, apoptosis and autophagy markers through IHC and IF. H&E-stained sections from lungs, liver, and primary tumor were evaluated by a board-certified veterinary anatomic pathologist (JBF) to quantify the number of metastatic lesions in a blinded fashion. For IHC, tumor slides were deparaffinized with xylene for 20 min and rehydrated using 100% and 90% ethanol for 20 min each. The slides were then washed twice with deionized water for 5 min. Antigen retrieval was performed in 10 mM citrate buffer by microwaving for 10 min followed by two washes with deionized water for 5 min each. The slides were then quenched in BLOXALL blocking solution (Cat# PK-8200, Vector Labs) for 15 min to block endogenous peroxidase activity and the slides were blocked with 2.5% normal horse serum for 30 min. Primary antibodies were diluted in 2.5% normal horse serum and added to the slides and incubated overnight at 4°C in a humidified chamber. The slides were then washed twice with 1% serum in PBS-T for 10 min each. For IF, the secondary antibodies were diluted in 1% serum in PBS-T and incubated for 2 hrs at room temperature. The slides were washed twice with 1% serum in PBS-T, and nuclear labelling was performed with DAPI containing anti-fade mounting medium. A coverslip was placed and sealed with nail polish. For IHC, the slides were incubated with prediluted biotinylated horse anti-mouse/rabbit IgG secondary antibody for 30 min and washed in PBS-T for 15 min. Then slides were incubated with VECTASTAIN elite ABC reagent for 30 mins and washed in PBS-T for 15 mins. DAB staining was performed until the intensities were reached and then the counterstaining was performed with hematoxylin (cat# 51275, Sigma). The slides were then washed with deionized water and dehydration was performed in 90% and 100% ethanol for 1 min each followed by 1 min in xylene incubation. The slides were then mounted using the mounting medium (Cat# 1442, ePredia). Stained slides were imaged using the Echo Revolution automated microscope (ECHO, USA) at 20× magnification, and quantified using ImageJ (RRID:SCR_003070) with the same threshold for each stain. The results were expressed as percent staining per visual field.

### Active RAS detection assay

RAS activation (RAS-GTP) levels were measured using the active RAS activation assay kit (Cell Signaling Technology, Cat# 8821). Lysates were prepared from cell lines or tumor tissues from various in vivo experiments by performing the steps provided by the manufacturer’s protocol. Tumors were lysed using the provided lysis buffer supplemented with protease and phosphatase inhibitors. A total of 1 mg/mL lysate in 1x lysis buffer was employed for the experiment. Equal amounts of protein were then incubated with the GST-Raf1-RBD protein, and the reaction mixture was loaded onto a RAS affinity resin to capture activated RAS. Following extensive washing to remove unbound proteins, bound protein was eluted in sample buffer and subjected to immunoblotting using a mouse RAS mAb (1:200 dilution) with gentle agitation overnight at 4 °C. The membrane was then probed with anti-mouse IgG, HRP-linked antibody (Cell Signaling Technology, Cat# 7076, RRID:AB_330924; 1:2000), and HRP-conjugated anti-biotin antibody (Cell Signaling Technology Cat# 7075, RRID:AB_10696897; 1:1000) to detect biotinylated protein markers. Activated RAS levels were measured using chemiluminescent reagents and quantified using the ImageJ system.

### Pharmacokinetics, tissue distribution and histopathological examination of ADT-030 in mice

Pathogen-free 8-week-old female C57BL/6J mice (Envigo#044; RRID:IMSR_ENV:HSD-044) were housed in the Biologic Research Laboratory at the University of South Alabama (U of SA), College of Medicine. Following acclimatization, mice were treated with ADT-030 at a dose of 100 mg/kg once daily for 14 days by oral gavage. Blood was collected at 0.5, 1, 2, 4, 8, and 24 hrs (n=4 per time point) following the last treatment into K_2_EDTA-microtainer tubes (BD Biosciences; Franklin Lakes, NJ; USA) to obtain plasma. Major organs (lungs, kidneys, spleen, heart, liver, brain, colon, and ovaries) were collected at 8 hrs (n=4). ADT-030 levels in plasma and organs were determined by LC-MS/MS. The study followed established guidelines and adhered to the approved protocol of the U of SA Institutional Animal Care and Use Committee (IACUC).

In another study, following acclimatization at the University of Alabama at Birmingham (UAB) animal facility, 5–6-week-old male C57BL/6J mice (The Jackson Laboratory #000664; RRID:IMSR_JAX:000664) were randomly assigned to two groups (n=5) and received ADT-030 (150 mg/kg) by oral gavage for 2 weeks. At the end of the treatment, blood was drawn for serum biochemical analysis, and mice were necropsied, organs were collected and fixed for histopathological analysis, and bone marrow smears were prepared for cytology. A board-certified veterinary anatomic pathologist (JBF) performed blinded assessment of organ viscera (heart, lung, kidney, liver, duodenum, pancreas, colon, spleen, thymus, testes, and brain) following standard procedures. The study followed established guidelines and adhered to the approved protocol of the UAB IACUC.

### Open field locomotor activity

Mice had *ad libitum* access to food and water throughout the experiments. Behavioral experiments were performed during the light cycle (between 8 a.m. and 6 p.m.). Before evaluation, mice were habituated for at least 30 min in the testing room. Mice were placed in an open-field arena (44×44×30 cm) in a dimly lit room (7 lx) and allowed to freely explore for 10 min as previously described ^35^. Locomotor activity was identified as the cumulative distance traveled during the entire 10 min. Statistical analyses were performed using GraphPad Prism (Version 10.4.1) using Student’s t-test for 2 groups and one-way ANOVA for comparing more than 2 treatment groups. Experimenters were blinded to treatment for all comparisons.

### Orthotopic grafting of PDAC cells in mice

*In vivo* studies followed established guidelines and adhered to the approved protocol of the UAB IACUC. 4–5-weeks-old male C57BL/6J mice (The Jackson Laboratory #000664; RRID:IMSR_JAX:000664) were subjected to isoflurane anesthesia, followed by an intra-abdominal incision to access the spleen and pancreas. A matrigel suspension (40 μL), containing KPC-*f*-luc (1 × 10^5^), 2838c3-*f*-luc (1 × 10^5^), or KPCML1-*f* -luc (1 × 10^5^) cells was injected into the pancreas. The skin and abdominal wall were then closed by suturing. Successful engraftment of the tumor cells was confirmed by PerkinElmer IVIS Lumina III *In Vivo* Imaging System (RRID:SCR_025239) one week later, and mice with tumors were randomized into four groups (n=5 per group) for KPC and 2838c3, and two groups for KPCML1 for monotherapy studies, and five groups for KPCML1 for chemotherapy combination study. Mice in PKC and 2838c3 studies were given oral dosages as follows: the first group received a vehicle, and the other three groups received ADT-030 at varying oral doses (50, 100, and 150 mg/kg). In the KPCM1 study, mice received vehicle or ADT-030 at 150 mg/kg. In the combination study with KPCML1 cells implanted, mice received vehicle, ADT-030 (150 mg/kg), or PF-2545920 (10 mg/kg, IP) daily for 4 weeks, gemcitabine (50 mg/kg, IP) plus nab-paclitaxel (30 mg/kg, IP), weekly twice (GPTx), and ADT-030 plus GPTx. Tumor tracking and response to therapy were monitored using D-luciferin injection and were conducted bi-weekly throughout the studies. The total luminescence from tumor-bearing regions was quantified using the Living Image *in vivo* imaging software. Body weights of the animals were measured twice a week. At termination, all animals were subjected to imaging of the whole body, followed by euthanasia, at which time tumors were collected, weighed, and used for subsequent experiments.

### Single-cell tumor processing

FFPE blocks of the tumor tissues from KPC orthotopic experiments treated with vehicle or ADT-030 (150 mg/kg) were used for sc-RNA sequencing, as per the standard protocol used at Admera Health (South Plainfield, NJ, USA). After sequencing, the data were analyzed by demultiplexing and aligned to the mouse reference genome (GRCm39) for gene expression quantification, and processed with Cell Ranger 9.0.1. The count matrices were then analyzed in Seurat (v.5.3.0) R package v.4.5.1. Cells with more than 200 genes and less than 5% mitochondrial content were kept for downstream analysis. After SC Transform analysis, PCA and UMAP were used for dimensionality adjustment and clustering. Differentially expressed genes (DEGs) were identified with FindallMarkers, and clusters were labeled using known markers.

### Tumor growth inhibition studies using PDAC PDX tumors

Mouse experiments were conducted using PDAC PDX models with KRAS^G12D^ and KRAS^G12C^ mutations as described previously ^36^. 4–5-week-old male NSG mice (The Jackson Laboratory# 005557; RRID:IMSR_JAX:005557) were utilized for the experiments. In brief, F1 generation tumors were cut into 2-mm × 2-mm fragments and subcutaneously implanted through a small incision made in the right flanks of NSG mice while they were anesthetized. Tumor size and body weight were monitored biweekly. Tumor volume was calculated using the formula: length × width^2^ × 0.5. Once tumors reached approximately 80–100 mm^3^, mice (n=5 per group) were randomly assigned into two groups. The first group received vehicle, and the second group received ADT-030 (150 mg/kg). A survival study was conducted for 70 days after 28 days of treatment. The UAB IACUC approved the experimental protocol for these mouse studies.

### Flow cytometry

Tumors derived from 2838c3-*f*-luc and KPC-*f*-luc PDAC cells implanted into the pancreas of C57BL/6J mice were digested using a solution containing 0.1 mg/mL DNase 1 and 1 mg/mL collagenase IV (Worthington Biochemical, Lakewood, NJ) in Hank’s Balanced Salt Solution (HBSS) at 37 °C with shaking for 45 min. Following digestion, the samples were rinsed, and enzymes were quenched with RPMI-1640-supplemented with 10% FBS, and a 70 μm strainer was used to filter them to produce single-cell suspensions. The separated cells were labeled for 60 min at 4 °C using primary antibodies conjugated to fluorophores and a live/dead dye (Supplementary Table 1). Cells were then rinsed and suspended in a FACS buffer (PBS + 2% FBS). After labeling the cell surface, the cells were fixed at room temperature in 4% paraformaldehyde or FoxP3 transcription buffer set (eBioscience# 00–5523-00) for 45 min, then washed with 1x Perm/Wash (BD, 554723) followed by resuspension in FACS buffer for data acquisition. Flow cytometry was performed. Data acquisition was conducted using a Symphony A5 flow cytometer, and analysis was performed using FlowJo.

### Statistical analysis

Statistical analyses and data visualization were done using GraphPad Prism (RRID:SCR_005375). The data are represented as means accompanied by either standard deviation (SD) or standard error of the mean (SEM). A repeated measures analysis of variance (ANOVA) or ANOVA with Bonferroni correction was conducted to evaluate and apply multiple corrections for assessing statistical significance between groups. A statistical significance threshold was set at p < 0.05.

## Results

### PDE10 overexpression in PDAC

**Figure 1:**
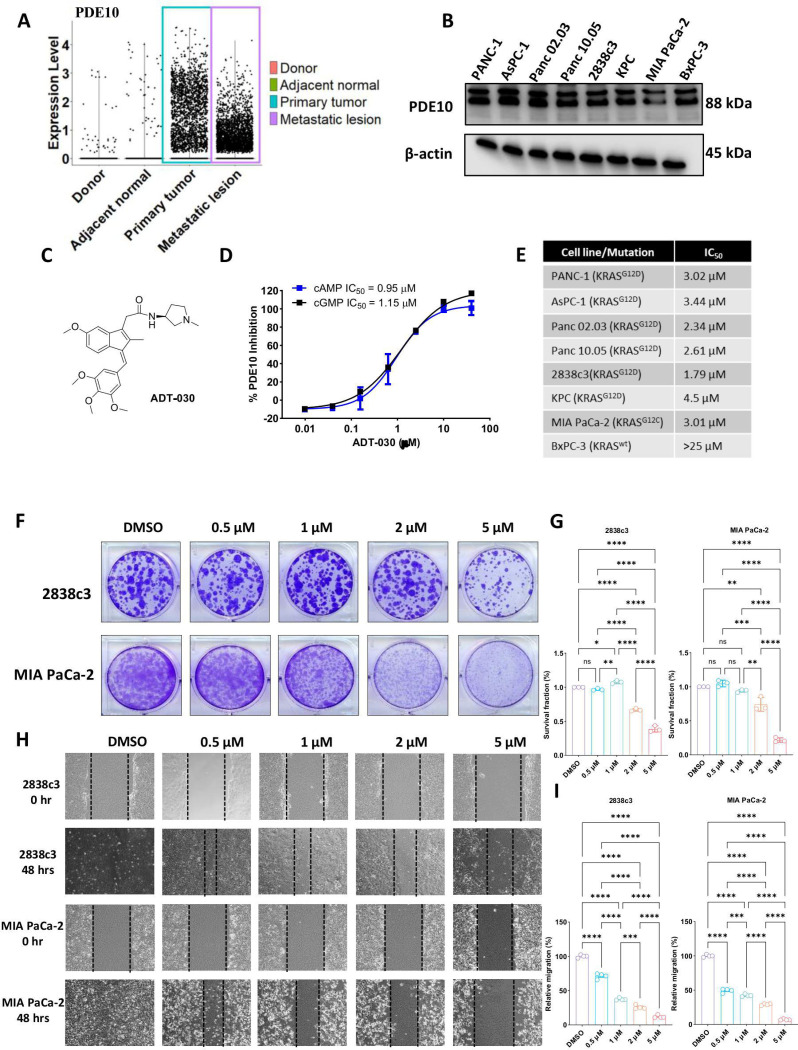
ADT-030 inhibits PDE10 at concentrations that inhibit proliferation, colony formation, and motility, while inducing apoptosis and cell cycle arrest of KRAS mutant PDAC cells. **A.** Violin plot of PDE10 expression in human donor, adjacent normal tissue, primary tumor and metastatic lesion as determined by sc-RNAseq analysis of human PDAC. **B.** Baseline expression of PDE10 across indicated PDAC cell lines. β-actin was used as a loading control. **C.** Chemical structure of ADT-030. **D.** ADT-030 inhibits the enzymatic activity of recombinant PDE10 using cAMP and cGMP as substrates. Data are expressed as mean ± SD, n = 2 samples/concentration. **E.** PDAC cell lines were treated with various concentrations of ADT-030 for 3 days followed by determining viable cell number using MTT assays. Relative percentage cell viability was plotted with respect to vehicle (DMSO) treated cells. The table lists the IC_50_ values for the PDAC cell lines treated with ADT-030. **F-G.** The indicated PDAC cell lines were treated with various concentrations of ADT-030 for 2–4 weeks, and long-term cell survival was measured using clonogenic assays. Representative images are shown in **F** and quantification plotted in **G. H.** Indicated PDAC cell lines were treated with vehicle or the indicated concentrations of ADT-030, and cell migration was analyzed using cell motility assays. Representative images under indicated treatment conditions for indicated PDAC cell lines are shown. **I.** Bar diagrams are presented to show relative migration (%) from the experiment presented in **H.** Data represents the mean ± SEM of three biological replicates. ns = not significant, *p < 0.05, **p < 0.01, ***p < 0.001, ****p < 0.0001. (one-way ANOVA).

To investigate the role of PDE10 in PDAC development, we used 10x Genomic sequencing and measured PDE10 expression from 229 patients across 12 different study groups. Employing sc-RNA seq data, we found that PDE10 mRNA is significantly enriched in primary tumors and metastatic lesions compared to tissues from normal donors and adjacent uninvolved tissues (Figure 1A). We then tested the PDAC cell lines used in our experiments and found that all expressed PDE10 protein (Figure 1B).

### ADT-030 inhibition and binding of PDE10

ADT-030 is an indene chemically related to the nonsteroidal anti-inflammatory drug, sulindac, designed to block cyclooxygenase (COX) inhibitory activity while targeting PDE10 (Figure 1C). The potency of ADT-030 to inhibit the enzymatic activity of recombinant PDE10 was determined by measuring cGMP and cAMP hydrolysis using a fluorescence polarization assay. ADT-030 inhibited cAMP and cGMP hydrolysis with IC_50_ values of 0.95 and 1.15 μM, respectively (Figure 1D). Molecular modeling studies using the PDE10 (2OUN) structure were performed by induced-fit molecular dynamics and simulation interaction analysis to identify a potential binding site on PDE10 for ADT-030. An optimal GLIDE docking score of −10.3 was calculated with ADT-030 bound in the PDE10 catalytic domain with the tri-methoxy benzylic moiety oriented toward the deep hydrophobic region of the pocket, while the more polar substituents were oriented towards the pocket entrance (Supplementary Figure 1A). The amide carbonyl and neighboring heteroatoms are predicted to form hydrogen bonds with His525 through bridging water molecules (Supplementary Figure 1B). Hydrophobic and aromatic contacts with residues Tyr524, Leu635, Phe639, Ile692, Tyr693, Phe696, Met714, Phe729, Val733, and Ala734 help to anchor the aromatic system within the binding site. These results suggest a favorable binding for ADT-030 in the PDE10 catalytic domain, which is supported by the docking score and a network of direct and water-mediated interactions, and consistent with a competitive mechanism of enzyme inhibition as previously reported for an analog, ADT-061 ^30^.

We next evaluated the antiproliferative activity of ADT-030 against a series of PDAC cell lines harboring various KRAS mutations, as well as wild-type RAS, by performing cell viability measurements using the MTT assay and determining potency (IC_50_) values. As shown in Figure 1E, ADT-030 inhibited the proliferation of all KRAS^G12D^ and KRAS^G12C^ mutant PDAC cell lines tested with IC_50_ values in the low micromolar range (1.8–4.5 μM). Notably, the KRAS wild-type PDAC cell line, BxPC-3, was found to be essentially insensitive to ADT-030, suggesting that ADT-030 selectively inhibits the proliferation of KRAS mutant PDAC cells (Figure 1E).

Experiments were also conducted to confirm that ADT-030 binds PDE10 in intact cells. In brief, HEK-293 cells expressing PDE10 Micro-Tag were treated with ADT-030. PDE10 thermal stability was measured by Micro-Tag enzyme complementation as described in the Materials and Methods section (Supplementary Figure 1C-F). The results revealed an EC_50_ value of 0.9 μM for ADT-030 to bind PDE10, which paralleled the potency ranges of ADT-030 to inhibit the enzymatic activity of PDE10 and the proliferation of PDAC cells.

### ADT-030 inhibits the clonogenicity and migration of KRAS mutant PDAC cells

A mouse PDAC cell line, 2838c3 (KRAS^G12D^ mutant), and a human PDAC cell line, MIA PaCa-2 (KRAS^G12C^ mutant), were selected to further study the anti-cancer activity of ADT-030. The long-term inhibitory effect of ADT-030 on cancer cell survival was evaluated in both PDAC cell lines by colony formation assays. ADT-030 treatment significantly reduced the number and size of colonies in both PDAC cell lines across a concentration range comparable to the potency (IC_50_) values to inhibit proliferation (Figure 1F-G). In addition, 2838c3 and MIA PaCa-2 cells showed significant impairment in motility after treatment with ADT-030 at non-cytotoxic concentrations (Figure 1H-I). Together, these results indicate that ADT-030 inhibits the proliferation, colony formation, and motility of PDAC cell lines harboring KRAS^G12D^ and KRAS^G12C^ mutations within the same concentration range as that is required to inhibit recombinant PDE10 and bind PDE10 in cells.

### ADT-030 induces apoptosis and G2/M cell cycle arrest in PDAC cells

To determine the effect of ADT-030 on apoptosis and cell cycle progression, the 2838c3 and MIA PaCa-2 PDAC cell lines were treated with ADT-030 for 24 and 72 hrs, respectively. Flow cytometry analysis of apoptosis as measured by Annexin V/PI staining showed that ADT-030 increased both early and late apoptotic cells at concentrations of 2 and 5 μM. In vehicle-treated 2838c3 cells, apoptotic cells comprised 1.6% of the population, whereas treatment with ADT-030 increased the percentage to 3.5% (2 μM) and ~8% (5 μM) (Supplementary Figures 2A and 2C). For MIA PaCa-2 cells, vehicle-treated cells had 2.3% apoptotic cells within the population, whereas ADT-030 treatment increased the number to 14.5% (2 μM) and 32% (5 μM) (Supplementary Figures 2B and 2D). Analysis of cell cycle distribution revealed that ADT-030 treatment increased the percentage of cells arrested in the G2/M phase in both PDAC cell lines (Supplementary Figures 2E-H).

### ADT-030 inhibits PDE10 and activates PKA/PKG signaling

**Figure 2.**
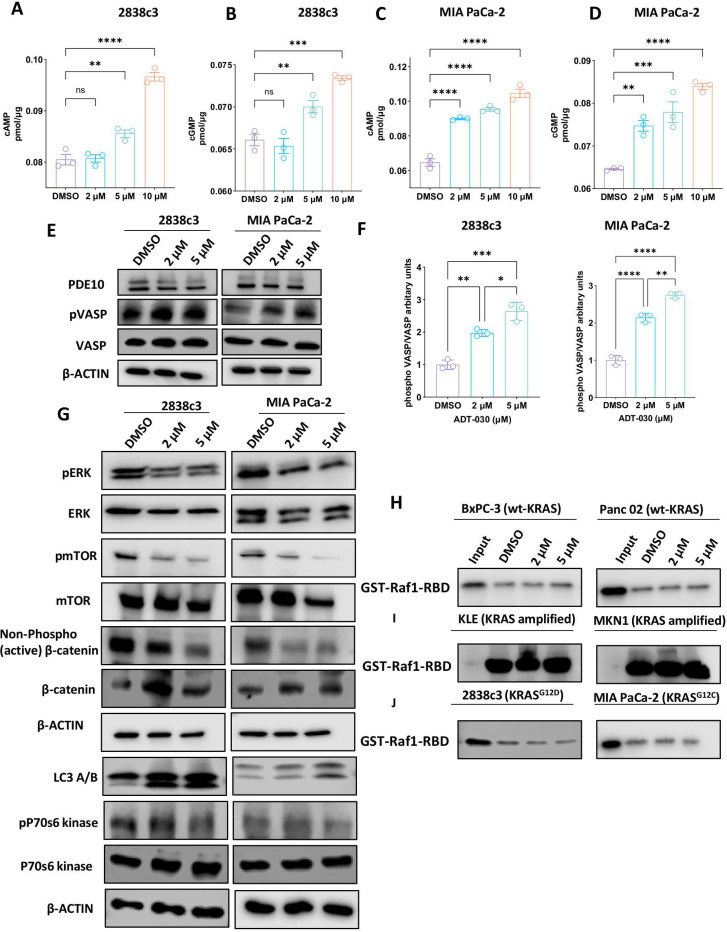
ADT-030 blocks PDE10 and activates PKA/PKG to reduce β-catenin levels and inhibit RAS signaling. **A-D.** ADT-030 increases cyclic nucleotide levels in a concentration-dependent manner in 2838c3 (**A-B**) and MIA PaCa-2 cells (**C-D**). **E-F.** Treatment with ADT-030 for 4 hrs did not affect the expression of PDE10 but induced VASP phosphorylation at serine 157 (PKA site) and serine 239 (PKG site) in a concentration-dependent manner in 2838c3 and MIA PaCa-2 cells. β-actin was used as a loading control. **G.** ADT-030 decreased phosphorylation of ERK, mTOR, and levels of active (oncogenic) β-catenin in 2838c3 and MIA PaCa-2 cells in a concentration-dependent manner. β-actin was used as a loading control. **H-J.** Indicated cell lines were treated with increasing concentrations of ADT-030 for 24 hrs and RAS pulldown was performed. Data represent the mean ± SEM of three biological replicates. ns = not significant, *p < 0.05, **p < 0.01, ***p < 0.001, and ****p < 0.0001 (one-way ANOVA).

Since PDE10 inhibition by ADT-030 is expected to increase intracellular levels of cAMP and cGMP, we measured both levels in 2838c3 and MIA PaCa-2 PDAC cell lines following treatment with ADT-030 by ELISA. Consistent with a PDE10 inhibitor, ADT-030 significantly increased the levels of cAMP and cGMP in a concentration-dependent manner in both PDAC cell lines. Notably, the effect was apparent at concentrations that paralleled the concentration range effective for inhibiting recombinant PDE10 and proliferation of both PDAC cell lines, as well as for inducing cell cycle arrest and apoptosis (Figure 2A-D). To determine if increased cyclic nucleotide levels by ADT-030 activated downstream protein kinases PKA and PKG, in 2838c3 and MIA PaCa-2 cells, we measured the phosphorylation of VASP (vasodilator-stimulated phosphoprotein), a known substrate for PKA and PKG ^37^. Consistent with a PDE10 inhibitor, ADT-030 increased VASP phosphorylation in both PDAC cell lines (Figure 2E-F), demonstrating that ADT-030 induced elevation of intracellular cAMP and cGMP levels results in the activation of PKA and PKG. Finally, it should be noted that ADT-030 treatment did not affect the expression of PDE10 (Figure 2E) or other cGMP and cAMP-degrading PDE isozymes, PDE3 or PDE4, respectively (Supplementary Figure 3A). We also determined if ADT-030 can activate canonical downstream signaling from PKA/PKG activation commonly reported in normal cells. Treatment of 2836c3 and MIA PaCa-2 cells with ADT-030 did not have any effect on level of activated phospho-CREB, VEGF-A, and Bcl-2 (Supplementary Figure 3B-C). This suggests that the activation of PKA and/or PKG by ADT-030 in PDAC cells may involve downstream targets unique to cancer cells.

### ADT-030 attenuates RAS and β-catenin signaling to promote apoptosis

Previous reports suggest that known PDE10 inhibitors and activation of PKG can phosphorylate the oncogenic pool of β-catenin in cell lines from various cancers ^25,27,29,30^. We therefore performed Western blot analysis to measure levels of the unphosphorylated (stable) form of β-catenin, representing the oncogenic pool of β-catenin required for TCF/LEF transcriptional activity in PDAC cells treated with ADT-030. ADT-030 treatment significantly reduced levels of the unphosphorylated form of β-catenin in 2838c3 and MIA PaCa-2 cell lines (Figure 2G). Previous research also suggested that PDE10 inhibitors and activation of PKG could suppress MAPK and AKT signaling in lung and ovarian cancer cells ^25,27^. Hence, we determined if ADT-030 has a similar effect in PDAC cells by measuring phosphorylated levels of ERK (pERK) and mTOR (pmTOR) within the MAPK and AKT signaling nodes, respectively. ADT-030 decreased pERK levels at its activating phosphorylation sites, Thr^202^ and Tyr^204^, as well as pmTOR at its activation site (Ser^2448^) (Figure 2G). These results suggest that the PDE10 inhibitory activity of ADT-030 can simultaneously suppress RAS and β-catenin signaling in PDAC cells.

To further study the effects of ADT-030 on RAS signaling, RAS-GTP pulldown assays were performed to measure activated RAS levels following the treatment of PDAC cells with ADT-030 for 24 hrs at concentrations of 2 and 5 μM. ADT-030 treatment did not reduce activated RAS levels in KRAS wild-type BxPC-3 and Panc 02 cells, as well as in KRAS amplified KLE (endometrial adenocarcinoma) and MKN1 (gastric adenocarcinoma) cancer cell lines (Figure 2H-I and Supplementary Figure 4A-D). Conversely, ADT-030 reduced activated RAS levels in 2838c3 cells expressing KRAS^G12D^ and MIA PaCa-2 cells expressing KRAS^G12C^ mutations at concentrations effective for inhibiting proliferation and PDE10 (Figure 2J and Supplementary Figure 4E-F). These results suggest that ADT-030 can inhibit activated RAS levels in KRAS-mutant PDAC cells but not in KRAS wild-type or KRAS amplified cells. This may explain the selective growth inhibitory activity of ADT-030 observed between RAS-mutated and RAS-wild-type PDAC cells.

We also analyzed the effect of ADT-030 treatment on autophagy in 2838c3 and MIA PaCa-2 PDAC cell lines, given previous studies reporting that RAS signaling and autophagy are interconnected and that RAS can modulate autophagy to promote tumorigenicity ^38^. Autophagy was assessed by Western blotting using LC3A/B as a marker. ADT-030 treatment increased the expression of LC3A/B, indicating its capacity to disrupt autophagic flux (Figure 2G). The cells were also treated with ADT-030 alone or in combination with hydroxychloroquine (HCQ), a known autophagy inhibitor. ADT-030 in combination with HCQ did not increase the levels of LC3A/B, suggesting that ADT-030, like HCQ, inhibits autophagic flux (Supplementary Figure 5A), and are consistent with a previously reported analog of ADT-030 ^39^. Furthermore, ADT-030 treatment reduced the expression of p70s6 kinase in 2838c3 and MIA PaCa-2 cells, which is associated with reduced migratory capacity (Figure 2G) ^40^.

### Hematologic, clinical chemistry, histopathologic and behavioral assessment of ADT-030 treated mice

**Figure 3.**
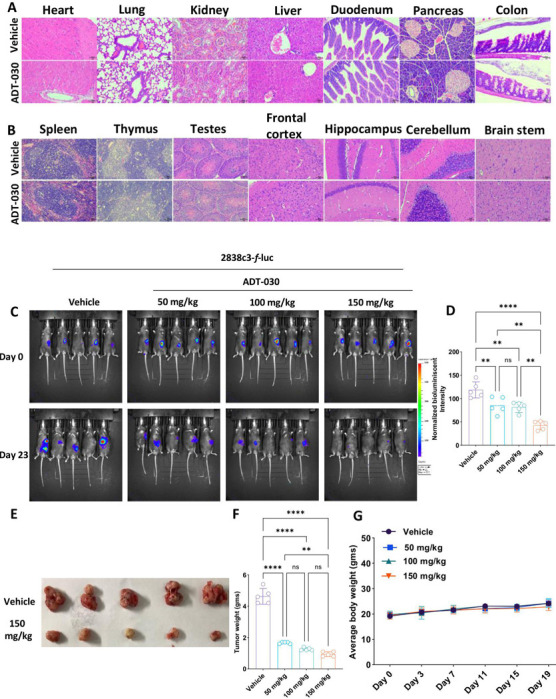
Effect of ADT-030 treatment on tumor growth and modulation of TME in 2838c3 cell-implanted C57BL/6J mice. **A-B.** Histopathological examination of vital organs (heart, lung, kidney, liver, duodenum, pancreas, colon, spleen, thymus, testes, and brain) from mice treated with ADT-030 at 150 mg/kg as assessed by H&E staining. **C.** 2838c3-*f*-luc cells were injected orthotopically into the pancreas of C57BL/6J mice. Representative bioluminescence images at the indicated time points are shown. **D.** Relative normalized whole-body bioluminescence intensities in mice under the indicated conditions (n = 5). Statistical significance was determined using one-way ANOVA. **E.** Images of the pancreatic tumors in the vehicle- and the 150 mg/kg ADT-030-treated groups at termination. **F.** Tumor weights at the end of the experiment for the indicated doses. **G.** Average body weights of mice treated with vehicle and ADT-030 (50, 100, and 150 mg/kg). Statistical significance was determined using one-way ANOVA. ns = not significant, **p < 0.01, and ****p < 0.0001.

Our next objective was to evaluate the tolerance of mice to ADT-030 treatment. Ten mice were randomly assigned to vehicle (n=5) or ADT-030 (150 mg/kg, n=5) treatment by oral gavage for two weeks. Complete blood counts (WBC, RBC, HGB, HCT, MCV, MCH, MCHC, RDW, PLT, MPV, neutrophils, lymphocytes, monocytes, eosinophils, and basophils) and serum biochemistry (albumin, ALT, ALP, amylase, total bilirubin, BUN, phosphorus, creatinine, glucose, electrolytes (calcium, sodium and potassium), total protein, and globulin were measured following two weeks of treatment (Supplementary Figure 6A-B). We observed no significant differences between the vehicle and ADT-030-treated mice, except for a slight reduction of total bilirubin levels in the treatment group. In addition, gross examination of multiple organs, including lungs, liver, kidney, pancreas, heart, duodenum, colon, spleen, thymus, brain, and testis, showed no histopathological abnormalities in ADT-030-treated mice compared with vehicle-treated mice (Figure 3A-B).

Sedation is a well-known side effect of conventional PDE10 inhibitors that were designed to cross the blood-brain barrier and developed for the treatment of CNS disorders (schizophrenia and Huntington’s disease). We therefore performed an open-field locomotor test to determine if ADT-030 causes sedation. These behavioral experiments revealed no significant differences between vehicle and ADT-030-treated mice, suggesting that ADT-030 does not cause sedation, a common side effect from previously developed PDE10 inhibitors (Supplementary Figure 7A).

### Pharmacokinetics and tissue distribution of ADT-030

A PK study was conducted in female C57BL/6J mice after oral gavage administration of 100 mg/kg ADT-030 once daily for 14 days. ADT-030 generated plasma levels that exceeded those required to inhibit PDE10 and PDAC cell growth *in vitro* (Supplementary Figure 7B). Plasma levels of ADT-030 reached a Cmax of 7 μM by 1 hr post-treatment and remained unchanged for an additional hour before decreasing by 4 hrs post-treatment to the level (5 μM). High levels of ADT-030 were also detected in various organs (lungs, kidneys, spleen, heart, liver, ovaries, and colon) 8 hrs after administration, but low levels were measured in brain (Supplementary Figure 7C). The low concentration of ADT-030 measured in the brain following oral administration likely account for the absence of sedation, a known side effect of conventional PDE10 inhibitors developed for the treatment of CNS disorders that were designed to cross the blood-brain barrier to achieve high concentrations in the brain ^41^.

### ADT-030 suppresses tumor growth in orthotopic PDAC model and reprograms the TiME

A mouse model of PDAC involving orthotopically implanted 2338c3-*f*-luc PDAC cells in the pancreas was initially used to assess the *in vivo* antitumor activity of ADT-030. Mice that established palpable tumors one week following implantation were randomized into four groups and treated by oral gavage administration with vehicle or ADT-030 at dosages of 50, 100, and 150 mg/kg once daily 5x/week for 23 days. Tumor progression was monitored using bioluminescence imaging (Figure 3C). All dosages of ADT-030 were effective, with the highest dose of ADT-030 tested showing tumor regression in all mice in the group. Quantitation of bioluminescence confirmed a significant reduction in tumor mass in ADT-030-treated mice compared to vehicle treatment (Figure 3D). Both tumor images and tumor weight measurements confirmed tumor shrinkage in ADT-030-treated groups in a dose-dependent manner compared to vehicle treatment (Figure 3E-F and Supplementary Figure 8A-B). ADT-030 treatment did not cause apparent systemic toxicity, as evidenced by no effect on body weight gain during treatment, suggesting the potential for greater efficacy at higher dosages or a more frequent dosing schedule (Figure 3G).

To study the immunomodulatory effects of ADT-030 relevant to PDAC, we performed multiparametric flow cytometry on orthotopic 2838c3 tumors from vehicle and ADT-030-treated mice. The analysis revealed that ADT-030 induced profound shifts in immune cell composition in the TiME, favoring a more immunostimulatory phenotype. ADT-030 treatment enhanced the immune cell infiltration within the TiME, resulting in a significant increase in overall populations of CD45^+^ leukocytes compared with vehicle treatment (Supplementary Figure 9A). Further characterization of the T-cell compartment revealed an increase in CD3^+^ T cells (Supplementary Figure 9B), observed with both CD4^+^ (Supplementary Figure 9C) and CD8^+^ T cells (Supplementary Figure 9D). Treatment with ADT-030 induced higher levels of several immune checkpoint markers, including CTLA-4 (Supplementary Figure 9E), PD-1 (Supplementary Figure 9F), LAG-3 (Supplementary Figure 9G), and TIGIT (Supplementary Figure 9H) in the total T cell populations. In another experiment involving the KPC-*f*-luc model, the immune checkpoint markers were differentially regulated with a decrease in CD8^+^ T cells, and an increase in CD4^+^ T cells. These results indicate that there was a concurrent adaptive immune regulatory response, likely representative of an acute but low, probably exhausted, T cell phenotype within the TiME. Apart from modulating the T cell compartment, ADT-030 also elevated NK cell infiltration within the TiME. Flow cytometry results revealed a pronounced increase in the NK1.1^+^ cell population in the tumors of the ADT-030-treated mice compared to vehicle-treated mice, indicating enhanced activation of the innate immune system (Supplementary Figure 9I). In addition to augmenting NK cell numbers, increased expression of immune checkpoint receptors was measured on NK cells by ADT-030 treatment. These observations were paralleled within the CD3^+^ T cell population, where ADT-030 enhanced infiltration of CD4^+^ and CD8^+^ T cells while upregulating CTLA-4, PD-1, LAG-3, and TIGIT (Supplementary Figure 9E-H). These data demonstrate that ADT-030 has the capacity to broadly remodel the immune landscape, attracting both adaptive and innate effector cells into the tumor while simultaneously engaging checkpoint regulatory pathways.

### ADT-030 alters RAS and β-catenin signaling in tumors

**Figure 4.**
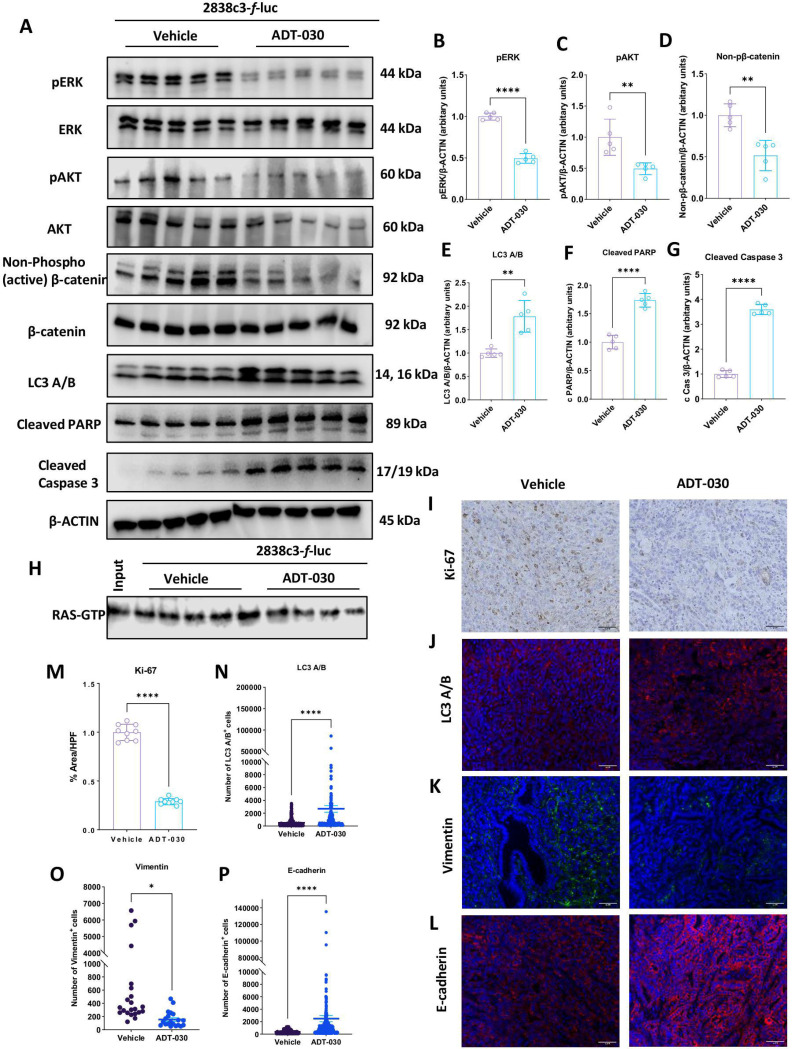
ADT-030 reduces β-catenin levels, inhibits RAS/AKT signaling, and induces autophagic cell death. **A.** Western blots showing levels of pERK, total ERK, pAKT, total AKT, non-phospho-β-catenin, total β-catenin, LC3A/B, cleaved PARP, and cleaved caspase 3 in 2838c3-*f*-luc tumors after vehicle or ADT-030 treatment. **B-G.** Bar graphs representing the quantifications of western blots from panel **A:** pERK (**B**), p-AKT (**C**), non-phospho-β-catenin (**D**), LC3A/B (**E**), cleaved PARP (**F**), and cleaved caspase 3 (**G**) in tumor tissues after ADT-030 vs. vehicle treatments. Welch *t*-test was used for statistical analysis. **H.** The inhibitory effect of ADT-030 on activated (GTP-bound) RAS in tumors after vehicle or ADT-030 treatments was assessed by RAS-RBD pull-down assay. **I.** Representative Ki-67 IHC results in tumors after vehicle or ADT-030 treatment. **J-L.** Representative IF images of LC3A/B **(J)**, vimentin **(K)**, and E-Cadherin **(L)** in tumors after vehicle or ADT-030 treatment. **M.** Bar graph representing the quantification of IHC staining for KI-67. **N-P.** Dot-plot graphs representing the IF quantifications of LC3A/B **(N)**, vimentin **(O)**, and E-Cadherin **(P)** in tumors after vehicle or ADT-030 treatment. Welch t-test was used for statistical analysis. ns, non-significant, *p < 0.05, **p < 0.01, and ****p < 0.0001.

To determine the activity of ADT-030 treatment to inhibit oncogenic signaling *in vivo*, 2838c3 tumors were harvested from mice treated with vehicle or ADT-030 and analyzed for key signaling and apoptosis markers. Western blot analysis revealed a marked decrease in pERK (Figure 4A-B) and pAKT (Figure 4A and 4C) levels with no effect on total ERK or AKT levels in tumors from ADT-030-treated mice, indicating the concurrent inhibition of the MAPK and PI3K/AKT pathways, respectively, reflective of upstream RAS inhibition. In addition, ADT-030 treatment reduced levels of the non-phosphorylated form of β-catenin, indicative of the stable pool of β-catenin driving transcription of proteins involved in oncogenesis, for example, from aberrant activation of WNT signaling (Figure 4A and 4D). Along with these alterations in key signaling node proteins, ADT-030 treatment also increased the expression of LC3A/B, consistent with *in vitr*o experiments, indicating that ADT-030 inhibits autophagic flux (Figure 4A and 4E). Increased levels of cleaved PARP (Figure 4A and 4F) and cleaved caspase 3 (Figure 4A and 4G) were also observed in the ADT-030-treated group, again consistent with *in vitro* experiments showing apoptosis induction by ADT-030 treatment. Also consistent with *in vitro* experiments, ADT-030 treatment reduced levels of activated (GTP-bound) RAS as measured by RAS-RBD pulldown assays (Figure 4H and Supplementary Figure 9J). IHC showed significantly reduced expression of the proliferation marker, Ki-67, in tumors from ADT-030-treated mice, corroborating the antiproliferative activity of ADT-030 as observed *in vitro* (Figure 4I and 4M). IF microscopy evaluation was used to analyze the treatment impact on autophagy and mesenchymal-to-epithelial transition (MET). Mice treated with ADT-030 at 150 mg/kg showed an increased level of LC3A/B in tumors, indicative of a disruption of autophagic flux (Figure 4J and 4N). ADT-030 also reduced vimentin expression (Figure 4K and 4O) and increased expression of E-cadherin (Figure 4L and 4P), which are associated with MET, and indicative of transforming cancer cells to a more normal epithelial phenotype with lower invasive capability.

### ADT-030 enhances antitumor immune responses and inhibits metastasis in an orthotopic PDAC model

**Figure 5.**
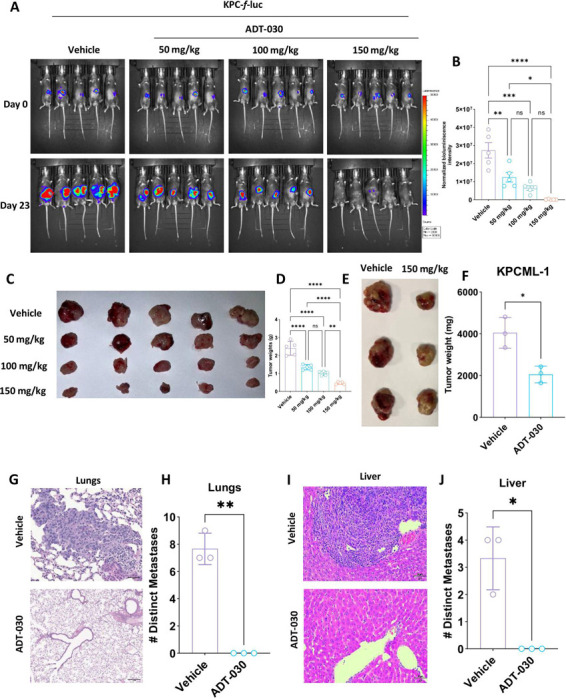
ADT-030 induces tumor growth arrest and regression in KPC and KPCML1 cells-implanted C57BL/6J mice and reduces liver and lung metastasis. **A.** KPC-*f*-luc cells were injected orthotopically into the pancreas of C57BL/6J mice. Representative bioluminescence images at the indicated time points are shown. **B.** Relative normalized whole-body bioluminescence intensities in mice under the indicated conditions (n = 5). Statistical significance was determined using one-way ANOVA. **C-D.** Tumor images after treatment with vehicle or ADT-030 at 50, 100, and 150 mg/kg (**C**) and bar graph representing tumor weights (**D**) from KPCML1 orthotopic model at termination. Statistical significance was determined using one-way ANOVA. **E-F.** Tumor images after treatment with vehicle or ADT-030 at 150 mg/kg (**E**) and bar graph representing tumor weights (**F**) from KPCML1 orthotopic model at termination. Statistical significance was determined using one-way ANOVA. **G.** Hematoxylin and eosin (H&E) staining of KPC-derived lungs after 4 weeks is shown. Representative images of H&E stained sections of metastasis in lung are displayed (20x). **H.** H&E staining of KPCML1-derived liver metastasis after 4 weeks is shown. Representative images of H&E staining sections from metastasis in lung are displayed (20x). **I-J.** Graph representing the quantification of lung **(I)** and liver **(J)** metastatic nodules in lung sections after vehicle or ADT-030 treatment from KPC and KPCML1 orthotopic experiments. Welch *t*-test was used for statistical analysis. ns, non-significant, *p < 0.05, **p < 0.01, ***p < 0.001, and ∗∗∗∗p < 0.0001

To confirm the anti-tumor effects of ADT-030 observed in the orthotopic 2838c3 tumors, we evaluated ADT-030 in the KPC orthotopic mouse model of PDAC to study specific immune cell subsets and functional T cell responses. To accomplish this, we implanted KPC-*f*-luc cells in C57BL/6J mice followed by treatment with ADT-030 (50, 100, and 150 mg/kg) or vehicle by oral gavage. Tumor growth was monitored by bioluminescence imaging on Day 0 and on Day 23 prior to euthanasia (Figure 5A). The KPC model recapitulated the major findings from the 2838c3 model. Normalized bioluminescence intensities showed statistically significant differences between the vehicle and treatment groups on Day 23 (Figure 5B). Both tumor size (Figure 5C) and tumor weights (Figure 5D) at the end of the experiment showed substantial shrinkage in a dose-dependent manner in the treated groups compared to the vehicle-treated group. We then performed IHC analysis for proliferation using the Ki-67 antibody in tumor sections from the KPC orthotopic model. Tumors treated with ADT-030 showed a marked decrease in the number of Ki-67 positive cells compared to the vehicle group, confirming the anti-proliferative activity of ADT-030 *in vivo* (Supplementary Figures 10A and 10E). To further substantiate the above findings, we performed immunofluorescence microscopy with the tumor tissues using autophagy (LC3A/B) and the MET markers, E-cadherin and vimentin. These analyses showed a significant increase in LC3A/B, suggestive of disrupted autophagy flux (Supplementary Figures 10B and 10F) and elevated levels of E-cadherin (Supplementary Figures 10C and 10G), along with a decrease in vimentin expression, reflective of MET (Supplementary Figures 10D and 10H).

### ADT-030 promotes anti-tumor immunity in a mouse model of PDAC

We then performed multiparametric flow cytometry on the excised KPC-*f*-luc tumors to assess the immune changes underlying ADT-030 treatment. A significant elevation in overall immune infiltration was observed, following ADT-030 treatment, as determined by increased frequency of CD45^+^ cells (Supplementary Figure 11A). Among the infiltrating pool of immune cells, there was an increase in αβ^+^ T cells (Supplementary Figure 11B), γδ^+^ T cells (Supplementary Figure 11C), TNK cells (Supplementary Figure 11D), and conventional NK cells (Supplementary Figure 11E), suggesting the activation of a broad-nature innate and adaptive immune response. The reduced expression of immune checkpoint molecules PD-1 (Supplementary Figure 11F) and CTLA-4 (Supplementary Figure 11G) on NK1.1^+^ cells, indicates NK cell exhaustion. In contrast, a marked increase in the frequencies of CD4^+^ T cells (Supplementary Figure 11H), as well as higher expression levels of PD-1, TIGIT, CTLA4^+^, PD-1^+^ CTLA4^+^ and FASr subsets (Supplementary Figure 11I), were observed in the CD4^+^ T cell compartment of ADT-030-treated tumors. The number of effector CD4^+^ T cells increased in the ADT-030-treated mice, signifying the involvement of helper T cell activation and functionality differences (Supplementary Figure 11J). Similar significant increases were present in overall CD8^+^ T cell numbers after ADT-030 treatment (Supplementary Figure 11K). In comparison, we measured decreased expression of immune checkpoint markers, including PD-1, CTLA-4, PD-1^+^CTLA-4^+^, and LAG-3, as well as lower expression of PD-1^+^CTLA-4^+^LAG-3^+^ triple-positive subsets (Supplementary Figure 11L) in the CD8+ T cell compartment. These data indicate a reversal of T cell exhaustion and re-engagement of cytotoxic potential. In addition, increased effector CD8^+^ T cells were observed with ADT-030 treatment, reflecting improved anti-tumor immunity (Supplementary Figure 11M).

Based on these results, we further explored the myeloid cell compartment in the TiME after ADT-030 treatment. We observed an enhanced influx of myeloid cells, as evidenced by increased total macrophages (F4/80^+^) (Supplementary Figure 11N). Another characteristic indicating myeloid infiltration was the increased expression of PD-L1 on macrophages after ADT-030 treatment, thereby enhancing antigen presentation and potential interaction with effector T cells (Supplementary Figure 11O). Phenotypic characterization of macrophages showed an increased M1-type characterized by MHC-II^+^CD86^+^ being more frequently expressed, while M2-like macrophages (CD206^+^) were less frequent from ADT-030 treatment compared to vehicle (Supplementary Figures 11P-Q). Additionally, an increased M1/M2 ratio was observed after ADT-030 treatment, signifying an enhanced immune-stimulatory TiME (Supplementary Figure 11R). Moreover, there was also an increase in the overall frequency of dendritic cells (DCs) post ADT-030 treatment (Supplementary Figure 11S). Both conventional subsets of dendritic cells, cDC1, and cDC2, were increased in frequency, suggesting enhanced antigen processing and presentation (Supplementary Figures 11T-U). This further rise in functional antigen-presenting cells, together with T and NK cell infiltration and activation, shows the wide immunomodulatory capacity of ADT-030 in remodeling of the pancreatic TiME toward an anti-tumor immune state.

### Single cell RNA-seq (scRNA-seq) of orthotopic KPC tumors treated with ADT-030

**Figure 6.**
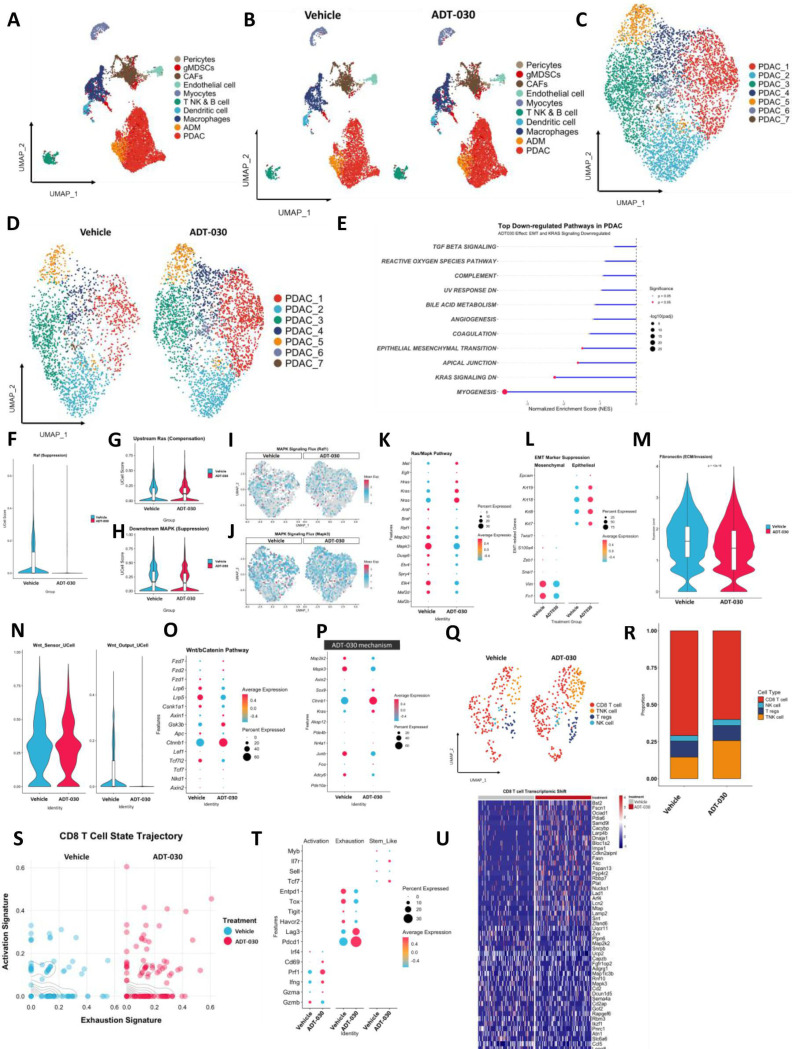
ADT-030 remodels tumor cell states and reinvigorates CD8 T cell in PDAC **A-B.** UMAP visualization of all single cells isolated from orthotopic PDAC tumors treated with either vehicle or ADT-030, colored by major cell types as indicated. **C.** UMAP reclustering showing 7 transcriptionally distinct PDAC cell types. **D.** Split UMAPs of PDAC cells from vehicle and ADT-030 tumors demonstrating treatment-associated shifts. **E.** Bar graph showing top significantly downregulated pathways in ADT-030 treated PDAC cells compared to vehicle as demonstrated by GSEA. **F-H.** Violin plots showing expression or module scores for RAF1 suppression **(F)**, upstream RAS **(G)**, downstream MAPK **(H)** related signatures in PDAC cells, including upstream WNT activation score **(F)**, a downstream Wnt target gene score **(G)**, and MAPK/ERK pathway score **(H)**, comparing vehicle and ADT-030. **I-J**. UMAP features plots demonstrating single-cell MAPK signaling flux for Raf1 **(I)**, and Map2k2 (ERK) **(J)** with corresponding dot plots summarizing average pathway activity (color scale) and fraction of PDAC cells expressing each gene set (dot size) in vehicle and ADT-030 tumors. **K-L.** Dot plots summarizing GSEA-derived pathway scores in PDAC cells highlighting RAS/MAPK pathway **(K)**, and reduced EMT **(L). M-N.** Violin plots showing stemness-associated EMT-related module scores for fibronectin in PDAC cells **(M)**, and WNT/β-catenin-dependent gene signatures **(N). O.** Dot plot of canonical WNT/β-catenin pathway genes across PDAC clusters. **P.** Dot plot of proposed mechanism-related genes illustrating transcriptional repression. **Q.** UMAPs of T and NK cell compartments in vehicle and ADT-030 tumors. **R.** Stacked bar plot quantifying the proportion of CD8 T cell, NK cell, T regs and TNK cell states among total T cells in vehicle *vs*. ADT-030. **S.** Scatter plot showing CD8 T cell state trajectory scores, with each point representing a single CD8 T cell positioned according to activation and exhaustion signature scores in vehicle and ADT-030 treatments. **T.** Dot plot summarizing the expression of representative activation, exhaustion, and stem-like genes in CD8 T cells. **U.** Heat map of CD8 T cell exhaustion-associated genes across individual CD8 T cells by trajectory state comparing vehicle and ADT-030 treated tumors depicting broad downregulation of exhaustion markers.

We also performed scRNA-seq on the resected tumors from this orthotopic KPC mouse experiment to further investigate the effects of ADT-030 on signaling pathways and immune microenvironment. A uniform manifold approximation and projection (UMAP) analysis was performed (Figure 6A), including 10 different cellular populations identified as pericytes, gMDSCs, CAFs, endothelial cells, myocytes, T NK and B cells, dendritic cells, macrophages, acinar to ductal metaplasia (ADM), and PDAC (Figure 6A-B). These populations were identified based on the expression of canonical marker genes for mature terminal lineages (Supplementary Figures 12A-B). We then identified 7 PDAC sub-clusters (Figure 6C-D) in which pathway analysis revealed that ADT-030-treated mice had significant downregulation in EMT, apical junction, KRAS signaling, and myogenesis signaling (Figure 6E). We then focused on MAPK signaling, as this pathway plays a major role in driving PDAC. In ADT-030-treated mice, there was a significant reduction in the expression of Raf1, a downstream mediator of activated RAS, suggesting the functional downregulation of the RAS-MAPK pathway in response to ADT-030 treatment (Figure 6F-H). Although an increase in upstream RAS signaling was observed, the MAPK signaling flux analysis revealed a significant reduction in the expression of Raf1 and Mapk3 (Figure 6I-J), suggesting that downstream RAS signaling was completely inhibited. Furthermore, deep analysis revealed a reduction in the expression of several MAPK pathway genes, including Map2k2, Mapk3, Dusp6, and Elk4 (Figure 6K). We then analyzed the EMT pathway and found the concurrent reduction in the expression of mesenchymal markers such as vimentin and fibronectin 1 (FN1) (Figure 6L-M). Next, we analyzed the impact of ADT-030 treatment on WNT signaling and found that WNT pathway markers were also suppressed (Figure 6N), including APC, AXIN2, Lrp5 and Lrp6 (Figure 6O). Mechanistic investigation confirmed that ADT-030 treatment reduced expression of several MAPK and WNT pathway genes, supporting the similar observations at the protein level (Figure 6P).

Next, we focused on identifying the role of ADT-030 on the immune microenvironment. To this end, we sub-clustered the UMAP into four groups, including CD8 T cells, TNK cells, Tregs and NK cells (Figure 6Q). A concurrent increase in the TNK cells was identified after ADT-030-treated mice, suggesting that ADT-030-treatment may enhance anti-tumor immune responses (Figure 6R). Although the total number of CD8 T cells was reduced, higher numbers of activated CD8 T cells were present after ADT-030-treated mice compared to vehicle-treated mice (Figure 6S). We also analyzed several markers of CD8 T cell activation, exhaustion, and stem-like properties. A significant increase in activation markers, including CD69, Prf1, IFNγ, and granzyme A (Gzma) was observed in ADT-030-treated mice. These findings show that ADT-030 increases CD8 T cell activation and enhances cytotoxic potential towards an effector state despite an overall reduction in total CD8 T cell numbers (Figure 6T). Evaluation of the transcriptomic signature of CD8 T cells clearly showed that ADT-030 treatment increased the expression of several early-activation genes, effector differentiation factors, cytotoxicity mediators, and chemokine ligands in ADT-030-treated mice, suggesting increased CD8 T cell functionality (Figure 6U).

**Figure 7.**
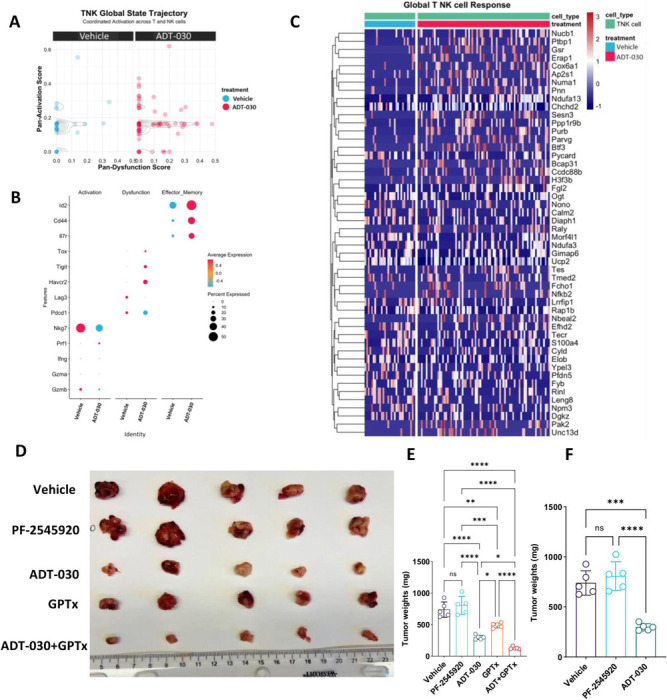
ADT-030 enhances global TNK activation and sensitizes chemotherapy to suppress PDAC tumor growth in vivo **A.** TNK global state trajectory plot displaying pan-activation *vs.* pan-dysfunction signatures in vehicle and ADT-030 treated tumors. **B.** Dot plot showing expression of activation, dysfunction, and effector genes in TNK cells from vehicle and ADT-030 treated tumors with dot size representing the percentage of expressing cells. **C.** Heat map of differentially expressed genes in TNK cells representing a global transcriptional shift toward an activated, cytotoxic program in ADT-030 treated tumors compared to vehicle. **D.** Representative images of tumors harvested from the mice treated with vehicle, PF-2545920, ADT-030, gemcitabine paclitaxel (GPTx), and the combination of ADT-030+GPTx. **E.** Quantification of tumor weights across all treatment groups showing tumor growth inhibition with ADT-030 and further significant reduction in ADT-030+GPTx combination groups compared to monotherapies and vehicle. **F.** Comparison of tumor weights between known PDE10 inhibitor (PF-2545920) and ADT-030 as monotherapy demonstrating superior efficacy of ADT-030. ANOVA and Welch t-test was used for statistical analysis. ns, non-significant, ∗p < 0.05, ∗∗p < 0.01, ∗∗∗p < 0.001, and ∗∗∗∗p < 0.0001.

ADT-030 treatment induced a similar but broader remodeling of the TNK compartment, extending the CD8 T-cell-specific effects to encompass both T and NK cells within the TiME (Figure 7A). In line with enhanced activation and reduced dysfunction of CD8 T cells, the TNK global state trajectory demonstrated that ADT-030 shifted TNK cells towards higher pan-activation scores with relatively lower pan-dysfunction scores compared to vehicle treatment, indicating a coordinated reinforcement of an activated, less dysfunctional state across cytotoxic lymphocytes. This was accompanied by increased expression and prevalence of key effector and activation genes such as Gzmb, Nkg7, and Prf1 (Figure 7B-C). Additionally, ADT-030 treatment induced a marked shift in NK cell functional state toward an activated phenotype compared to vehicle treatment (Supplementary Figure 12C). NK cell trajectory analysis revealed that ADT-030-treated mice showed higher activation and lower dysfunction signatures, indicating coordinated enhancement of activation programs. Concordantly, dot-plot analysis revealed increased expression and prevalence of activation and maturation markers, including Zeb2, Bcl2, Klrg1, Itgam, Cd160, Havcr2, Prf1, IFNγ, and Gzmb (Supplementary Figures 12D-E). Together, these results demonstrate that ADT-030 enhances cytotoxic lymphocyte activation within the TiME, driving CD8 T and TNK compartments towards a sustained, less dysfunctional effector state to enhance anti-tumor immunity.

### Effects of ADT-030 on metastasis

To investigate the potential of ADT-030 to block metastasis, we used an established metastatic PDAC cell line, KPCML1, derived from the KPC mouse model ^42^. KPCML1 cells have a high propensity for liver and lung metastasis, representative of patients with metastatic pancreatic cancer ^42^. Using similar tumor inoculation methods and treatment (vehicle *vs*. ADT-030 at 150 mg/kg daily) as described in the Materials and Methods section, we examined the impact of ADT-030 treatment on mice orthotopically implanted with KPCML1 PDAC cells. On day 23 after tumor implantation, ADT-030 treatment decreased the size and weight of the primary orthotopic tumor compared to vehicle treatment (Figure 5E-F). Luciferase levels were measured using *ex vivo* imaging, which revealed that while mice in the vehicle group implanted orthotopically with KPCML1 cells developed liver and lung metastasis, there was a complete absence of liver (Supplementary Figures 13A-B) and lung (Supplementary Figures 13C-D) metastasis in mice treated with ADT-030. We also analyzed lung and liver sections histologically by H&E staining, which confirmed metastasis in the vehicle group and supported the observed anti-metastatic activity of ADT-030 (Figure 5G-J).

### ADT-030 enhances the antitumor efficacy of chemotherapy in orthotopic PDAC models

KPCML1 cells were implanted orthotopically and treated with vehicle, ADT-030 (150 mg/kg), standard-of-care chemotherapy (a combination of gemcitabine, 50 mg/kg and nab-paclitaxel, 10 mg/kg, GPTx), or a combination of ADT-030 with GPTx. ADT-030 produced a better therapeutic effect than chemotherapy as indicated by tumor size and tumor weight measurements (Figure 7D-F). Interestingly, the combination of ADT-030 with chemotherapy showed better efficacy compared to chemotherapy or ADT-030 alone, demonstrating that ADT-030 has the potential to enhance standard-of-care chemotherapy efficacy for the treatment of PDAC.

### ADT-030 has increased potency and improved therapeutic window compared to other PDE10 inhibitors

PF-2545920 is a known PDE10 inhibitor developed for CNS disorders such as schizophrenia and Huntington’s disease. The potency IC_50_ values for PF-2545920 to inhibit the proliferation of MIA PaCa-2 and 2838c cells were measured to be 25.02 and 24.1 μM, respectively (Supplementary Figure 14A) compared with ADT-030 having IC_50_ values of 3.01 and 1.79 μM, respectively (Figure 1E). Similarly, colony formation assays revealed that PF-2545920 had IC_50_ concentrations exceeding 25 μM (Supplementary Figure 14B-C) whereas ADT-030 showed significant inhibition of colony formation at 5 μM (Figure 1G-H).

We then compared PF-2545920 with ADT-030 in the KPCML1 orthotopic mouse model of PDAC. Mice were treated with vehicle, ADT-030 (150 mg/kg), or PF-2545920 (10 mg/kg) in which each were once daily by oral gavage. PF-2545920 failed to show antitumor activity, while ADT-030 significantly inhibited tumor growth as evidenced by tumor images and measurement of tumor weight (Figure 7D-F). Additionally, we evaluated the effects of PF-2545920 and ADT-030 treatments on mice using open-field locomotor tests. There were no significant differences in behavior or mobility between ADT-030 and vehicle-treated mice, whereas PF-2545920-treated mice displayed significantly reduced mobility throughout the test, reflective of sedation, a known side effect of conventional PDE10 inhibitors (Supplementary Figure 15A). These data led us to conclude that ADT-030, but not PF-2545920 displays antitumor activity without causing sedation, which likely reflects differences in brain and systemic levels between ADT-030 and known PDE10 inhibitors.

(Supplementary Figure 15A). This data led us to conclude that ADT-030 displays superior antitumor activity compared to a known PDE10 inhibitor without causing sedation.

### ADT-030 suppresses tumor growth and induces prolonged responses in KRAS^G12D^ and KRAS^G12C^ PDAC PDX models

**Figure 8.**
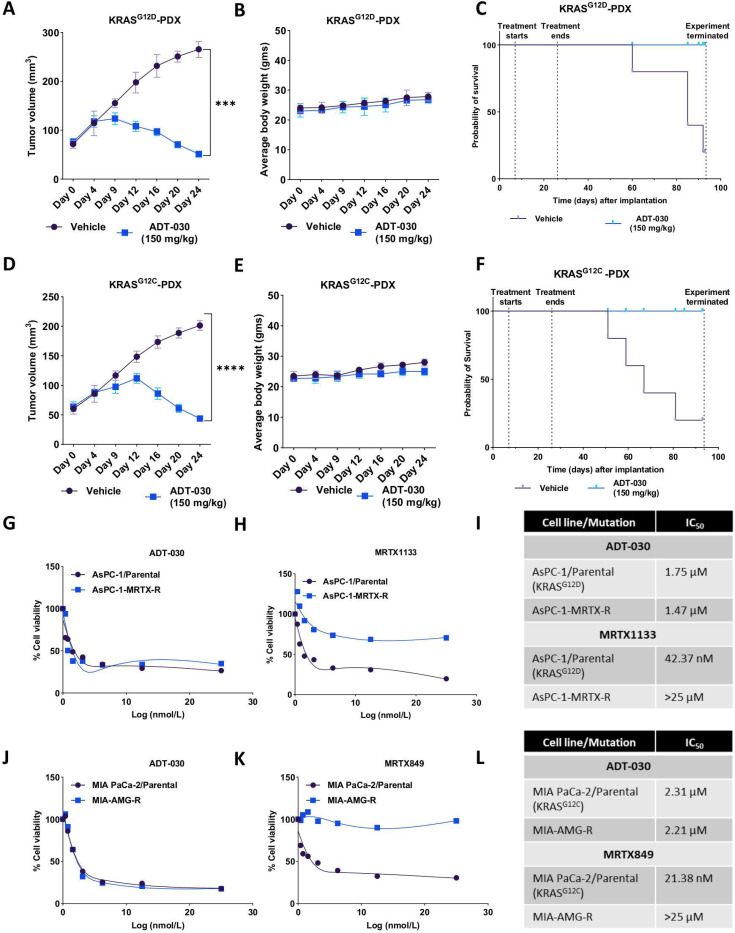
ADT-030 induces tumor regression and extends survival in KRAS^G12D^ and KRAS^G12C^ PDX models with potential to escape resistance to KRAS^G12D^ and KRAS^G12C^ inhibitors. **A.** Tumor growth in NOD.Cg-Prkdc^scid^ Il2rg^tm1Wjl^/SzJ mice implanted with KRAS^G12D^ PDX and treated with vehicle or 150 mg/kg ADT-030. **B.** Average body weights of mice treated with vehicle or ADT-030. **C.** Survival curves of mice after the treatment with the vehicle or ADT-030. **D.** Tumor growth in NOD.Cg-Prkdc^scid^ Il2rg^tm1Wjl^/SzJ mice implanted with KRAS^G12C^ PDX and treated with vehicle or ADT-030. **E.** Average body weights of mice treated with vehicle or ADT-030. **F.**Survival curves of mice after the treatment with the vehicle or ADT-030. **G-H.** The indicated PDAC cell lines were treated with various concentrations of ADT-030 (G) or MRTX1133 (**H**) and subjected to MTT assays. Relative percentage cell viability was plotted relative to vehicle treated cells. **I.** Table showing the IC_50_ values for the cell lines used in panels G-H. **J-K.** The indicated PDAC cell lines were treated with various concentrations of ADT-030 (**J**) or MRTX849 (**K**) and subjected to MTT assays. Relative percentage cell viability was plotted relative to vehicle treated cells. **L.** Table listing the IC_50_ values for each PDAC cell line shown in panels J-K.

To further evaluate the efficacy of ADT-030 in suppressing PDAC tumor growth *in vivo*, we used two clinically annotated subcutaneously implanted PDX models of PDAC harboring KRAS^G12D^ and KRAS^G12C^ mutations. ADT-030 was administered orally at a dose of 150 mg/kg for 23 days, once daily, 5x/week. Tumor dimensions and body weight were measured twice/week. The results displayed a strong anti-tumor response in both KRAS^G12D^ (Figure 8A) and KRAS^G12C^ PDX models (Figure 8D) with no apparent systemic toxicity in terms of reduction in body weight (Figure 8B and 8E). The treatment was stopped after four weeks, and mice were monitored for tumor recurrence and survival. Mice treated with ADT-030 did not develop tumor regrowth over 70 days of follow-up (Figure 8C and 8F), while vehicle-treated mice progressively died during the post-treatment period. These data demonstrate both robust and durable antitumor activity of ADT-030 in clinically relevant PDX models of PDAC.

### ADT-030 suppresses the growth of KRAS^G12D^ and KRAS^G12C^ - resistant PDAC cells

Finally, we tested the anti-proliferative activity of ADT-030 in KRAS^G12D^ and KRAS^G12C^ resistant cell lines grown *in vitro*. AsPC-1 cells resistant to MRTX1133 (AsPC-1-MRTX-R) and parental cells were treated with ADT-030 or MRTX1133, a KRAS^G12D^ inhibitor ^43^. Cell viability measurements using the MTT assay revealed that ADT-030 showed comparable antiproliferative activity in both parental (IC_50_ = 1.75 μM) and AsPC-1-MRTX-R cells (IC_50_ = 1.47 μM) (Figure 8G-I), whereas MRTX1133 inhibited the proliferation of parental AsPC-1 cells (IC_50_ = 43.74 nM), but not of the AsPC-1-MRTX-R cells (IC_50_ > 25 μM), confirming that these cells are resistant to MRTX1133. We also determined if this effect is sustained with longer treatment durations by performing colony formation assays. The results showed activity of ADT-030 similar as proliferation assays, where ADT-030 inhibited colony formation in both parental and AsPC-1-MRTX-R cells, while MRTX1133 inhibited colony formation only in parental cells (Supplementary Figures 16A-B). We also treated MIA PaCa-2^G12C^ parental and MIA PaCa-2 resistant to MRTX849 and AMG-510 (MIA-AMG-R) cells with ADT-030 and MRTX849, a KRAS^G12C^ inhibitor ^44^. ADT-030 inhibited the proliferation of both MIA PaCa-2 parental and MIA-AMG-R cells with comparable potency. In contrast, MRTX849 inhibited the proliferation of MIA PaCa-2 parental cells, but not MIA-AMG-R (Figure 8J-L). Similar results were found in colony formation assays, whereas ADT-030 reduced the number and size of colonies in both MIA PaCa-2 parental and MIA-AMG-R cells, while MRTX849 reduced the colony formation only in MIA PaCa-2 parental cells (Supplementary Figures 16C-D). Together, these results show that ADT-030 exhibits a broad spectrum of RAS inhibitory activity and has the potential to escape acquired resistance that limits the efficacy of mutant-specific KRAS^G12D^ and KRAS^G12C^ inhibitors.

## Discussion

Aberrant activation of MAPK/AKT signaling from KRAS mutations, along with the activation of WNT/β-catenin mediated transcription, plays a major role in driving cancer cell proliferation, survival, and metastasis in PDAC, and other cancers, including colorectal, liver, lung, and breast cancer ^45–48^. KRAS is mutated in over 90% of PDAC, while mutations or increased expression of β-catenin or other pathway components (e.g., Wnt ligands, FZD receptors, LRP co-receptor, APC, Axin) are also observed in a high percentage of patients with PDAC and other cancers ^49,50^. Compensatory or cooperative interactions between these signaling pathways likely also contribute to aggressiveness of the disease as well as therapy resistance ^51^. Phosphodiesterase isozymes have been previously studied in the context of cancer, but no particular isozyme has been targeted by an inhibitor, and no PDE inhibitor has received FDA approval for the treatment of cancer ^22^. Recently, several publications have reported that isozyme-specific PDE10 inhibitors or genetic silencing of PDE10 can block RAS and β-catenin by activating PKG ^25,27,29,30^. Similarly, cAMP-activated PKA can inhibit signaling downstream of RAS by disrupting interaction with Raf1 ^52^. Our data revealed increased expression of PDE10 in PDAC cells as compared to adjacent normal pancreatic tissue, which provided an initial rationale to targeti this pathway for the treatment of PDAC. ADT-030 is a non-COX inhibitory sulindac derivative and a second-generation analog of ADT-061 (aka MCI-030), previously reported to selectively inhibit PDE10 and the proliferation of colorectal cancer and ovarian cancer cell lines ^25,30^. In these studies, PDE10 knockdown resulted in reduced sensitivity of the cancer cells to ADT-061, as well as known PDE10 inhibitors, which confirmed the selectivity of this class of agents to PDE10 and suggested that PDE10 is a understudied vulnerability of cancer cells. Molecular docking simulations and cellular thermal stability assays presented in this study provide structural insight into the interaction between ADT-030 and PDE10 and confirmation of target engagement, respectively. These findings support an mechanism of action for ADT-030 involving PDE10 inhibition, elevation of cyclic nucleotides, and protein kinase activation and support future research are needed to further study the oncogenic role of PDE10.

Here, we show that ADT-030 inhibits the enzymatic activity of recombinant PDE10 and activates cyclic nucleotide signaling in PDAC cells at concentrations that selectively inhibit the proliferation of KRAS mutant PDAC cells. Of clinical relevance, we found that ADT-030 also inhibits the proliferation of PDAC cells that develop resistance to KRAS inhibitors and can enhance the efficacy of standard-of-care chemotherapy, suggesting that ADT-030 has the potential to be a front-line treatment for patients with PDAC. ADT-030 is distinct from other KRAS inhibitors, FDA-approved or in development, by its capacity to escape resistance, which we attribute to cell cycle arrest and the induction apoptosis, resulting from the dual blockage of β-catenin and RAS signaling. The observed inhibition of MAPK and AKT/PI3K pathways by ADT-030 is particularly significant for PDAC treatment, as both pathways are known for their extensive crosstalk and compensatory activation to drive cancer cell proliferation and survival ^53,54^. Compensation from β-catenin may also contribute to resistance to monospecific inhibitors of KRAS or β-catenin where by dual blockage of RAS or β-catenin pathways through PDE10 inhibition may prevent the development of resistance to KRAS inhibitors, FDA-approved or in development ^55^. To corroborate these findings using gene expression profiling, we evaluated tumors excised from mice treated with ADT-030 or vehicle using single-cell transcriptomics. The results confirmed suppressive effects of ADT-030 on key oncogenic signaling pathways, including RAS-MAPK, EMT, and WNT, as evidenced by reduced expression of Raf1, Mapk3, Map2K2, vimentin, FN1, APC, and Axin2. We also conducted assays on RAS activation in RAS wild-type and KRAS mutant PDAC cell lines and found that ADT-030 selectively inhibited RAS activation in KRAS mutant PDAC cell lines. This interesting observation needs further study to understand the differential effects of PDE10 inhibition and impact of cyclic nucleotide signaling in KRAS mutant versus RAS wild-type PDAC cells.

ADT-030 is orally bioavailable with attractive drug-like properties and appears to be well tolerated at dosages that exhibit robust and durable antitumor activity. We found that ADT-030 inhibits both primary tumor growth and metastasis without discernible toxicity in several mouse models of PDAC, including PDX and orthotopic models. ADT-030 also potentiated the efficacy of standard-of-care chemotherapy regimens for PDAC. These findings support the rationale for developing ADT-030 as a front-line treatment for PDAC as a monotherapy or in combination with standard-of care chemotherapy.

PDE10 inhibitors have been previously developed for the treatment of CNS disorders such as schizophrenia and Huntington’s disease. We therefore compared ADT-030 to the known PDE10 inhibitor, PF-2545920, and found that ADT-030 displayed appreciably greater potency than PF-2545920 to inhibit PDAC cell proliferation *in vitro*. This observation suggests that although PDE10 is a cancer target, PF-2545920 has low potency to inhibit cancer cell proliferation, which may be attributed to compensation by other co-expressed PDE isozymes (e.g., PDE5). When compared to ADT-030 *in vivo*, PF-2545920 failed to demonstrate anti-tumor activity. In addition, ADT-030 displayed improved tolerance without the side effects (sedation) observed with PF-2545920 ^56^.

PDAC is also known to be associated with an immunosuppressive TiME, a major factor responsible for resistance to immunotherapy ^57,58^. The immune infiltration in PDAC is characterized by the abundance of immune suppressive cells and the lack of anti-tumor immune cells^59^. Activation of the immune system by ADT-030 treatment is another intriguing and clinically relevant finding as we report. ADT-030 treatment increased CD4^+^ and CD8^+^ T cells, as well as NK cell infiltration, resulting in a shift towards M1-like macrophage polarization. The inhibition of expression of CTLA-4, PD-1, and LAG-3 on CD8^+^ T cells by ADT-030 treatment suggests that ADT-030 alleviates T cell exhaustion and reestablishes cytotoxic T cell function within the TiME ^60^. Aside from maintaining antitumor T cells, ADT-030 also enhanced myeloid cell infiltration by increasing the number of total macrophages (F4/80^+^) within the TiME. In particular, these macrophages displayed enhanced antigen-presenting potential as evidenced by increased expression of PD-L1, crucial for effector T cell interaction. In addition, phenotypic analysis revealed a shift in macrophage polarization toward a pro-inflammatory, M1-like phenotype (MHCII^+^ CD86^+^), which was accompanied by a decrease in M2-like macrophages (CD206^+^) ^61^. These results were further supported by the identification of a high M1/M2 ratio. In addition to macrophages, ADT-030 treatment led to an increase in conventional dendritic cells (cDC1 and cDC2), contributing to activated T and NK cells ^62^. Our single-cell RNA sequencing data demonstrated that the cytotoxic lymphocyte compartment is reprogrammed, with CD8 T cells and TNK cells exhibiting enhanced activation, effector function, and reinvigoration. Activation of cytotoxic genes (IFNγ, Gzma, and Prf1) and activation markers (CD69 and Cxcr3), alongside modulation of exhaustion pathways (Lag3 and CTLA4), indicates that ADT-030 not only suppresses tumor cell proliferation but also potentiates anti-tumor immunity. These convergent tumor-cell and immune-cell effects provide a strong mechanistic rationale for evaluating ADT-030 in combination with immune checkpoint blockade or other immunomodulatory strategies, with the goal of converting immunologically cold PDAC into a more treatment-responsive state. This will be a future direction for preclinical studies combining ADT-030 with immune checkpoint blockade and possible clinical trials, given the limitations of immunotherapy for the treatment of PDAC.

While mutant-selective KRAS^G12C^ and KRAS^G12D^ inhibitors have demonstrated promising efficacy for KRAS mutant cancers ^63,64^, acquired resistance remains a major clinical limitation. Various mechanisms of resistance have been reported, including secondary RAS mutations, activation of co-expressed RAS wild-type isozymes, and compensatory receptor tyrosine kinase mutations, all of which frequently emerge in recurrent tumors and contribute to treatment failure, disease relapse, and death of the patient ^65^. In the current study, we investigated whether ADT-030 may be less susceptible to the same mechanisms of resistance that limit the efficacy of KRAS^G12C^ and KRAS^G12D^ inhibitors using PDAC cell lines developed to be resistant to such drugs. Strikingly, ADT-030 demonstrated potent anti-proliferative activity in both MRTX1133 and MRTX849 resistant PDAC cell lines, highlighting its broad-spectrum pan-RAS inhibitory activity and its ability to bypass diverse mechanisms of acquired resistance to allele-specific KRAS inhibitors.

The therapeutic potential of ADT-030 is supported by the antitumor results observed in clinically and genetically relevant PDAC PDX models harboring KRAS^G12D^ and KRAS^G12C^ mutations as well as several orthotopic mouse models. In these experiments, efficacy and tolerability of ADT-030 were assessed following oral administration at a dose of 150 mg/kg for 4 weeks. This dosage caused no discernible toxicity and is a human equivalent dosage of 12 mg/kg, or 840 mg once daily for a 70 kg human, a moderate dose for many drugs. Treated mice showed tumor regression and no tumor regrowth for over 70 days after stopping treatment, highlighting the durability in maintaining long-term tumor control. These findings provide compelling evidence of robust and clinically relevant antitumor activity of ADT-030 by inhibiting PDE10 that support IND-enabling studies. This activity and capacity of ADT-030 to simultaneously block RAS and β-catenin signaling also supports further mechanistic studies of the oncogenic role of PDE10 (Supplementary Figure 17). Future studies focusing on identifying the activity of ADT-030 in genetically engineered mouse models as a monotherapy and in combination with immune checkpoint inhibitors will further help in the translation of this agent to the clinic. In conclusion, the ability of ADT-030 to inhibit PDE10 and block multiple aspects of malignant progression, including cancer cell proliferation, survival, and metastasis, as well as creating a more favorable TiME, while having the potential to escape resistance that limits the efficacy of other RAS inhibitors, makes ADT-030 a highly desirable drug development candidate for clinical trials in patients with metastatic PDAC and other RAS-driven malignancies.

## Supplementary Material

Supplement 1 **Supplementary Figure 1**: **A.** Docking of ADT-030 to PDE10 (2OUN) resulted in an optimal docking score of −10.325. Representative surface rendering of 2OUN with electrostatic potential mapped onto the surface shows ADT-030 bound in the PDE10 catalytic pocket. ADT-030 is rendered as a ball-and-stick representation. Pink and grey spheres illustrate magnesium and zinc ions in the pocket, respectively. **B.** Interaction diagram showing molecular interactions of ADT-030 with PDE10. **B**inding affinity of ADT-030 to PDE10 as determined by treating HEK293 cells (45 min) expressing PDE10-Micro-Tag with ADT-030. **C.** Western blot analysis showing PDE10 expression in HEK293 cells transfected with PDE10-Micro-Tag and detected using anti-Micro-Tag antibody. **D.** Quantification of Micro-Tag enzyme complementation in cells transfected with PDE10-Micro-Tag construct vs untransfected HEK293 cells. **E.** PDE10 thermal curve yielding a Tagg_50_ of 44°C, providing the fixed challenge temperature to determine ADT-030 binding to PDE10 in transfected HEK293 cells. **F.** ADT-030 binding to PDE10 in transfected HEK293 cells. The curve is graphed as the average of two replicates ± SEM.

Supplement 2 **Supplementary Figure 2**: **A-B.** Representative FACS analysis showing ADT-030 induced apoptosis in 2838c3 **(A)**, and MIA PaCa-2 **(B)** cells after treatment with ADT-030 (2 and 5 μM) or vehicle for 24 hrs. After double-staining with annexin V and PI, cells were subjected to flow cytometry analysis. **C-D.** The indicated PDAC cell lines were treated with ADT-030 at varying concentrations for 24 hrs, and apoptosis was measured following annexin V/propidium iodide labeling. **E-F.** Representative DNA histogram showing cell cycle arrest in 2838c3 **(E)**, and MIA PaCa-2 **(F)** cells after treatment with the indicated concentrations of ADT-030 or vehicle controls for 72 hrs. **G-H.** Flow cytometry analysis of cell cycle distribution in 2838c3 **(G)** and MIA PaCa-2 **(H)** cells treated with vehicle or ADT-030 at the indicated concentrations. Data represents the mean ± SEMr of three biological replicates. ns = not significant, *p < 0.05, **p < 0.01, ***p < 0.001, ****p < 0.0001. (one-way ANOVA).

Supplement 3 **Supplementary Figure 3**: **A-C.** Western blot analysis showing the expression of indicated PDE isozymes, pCREB CREB, VEGFA, and Bcl-2 in 2838c3 and MIA PaCa-2 cells treated with DMSO, and varied time points/concentrations of ADT-030.

Supplement 4 **Supplementary Figure 4**: **A-F.** Quantification of RAS-GTP activation in BxPC-3, Panc 02, KLE, MKN1, 2838c3, and MIAPaCa-2 cells treated with either DMSO or increasing concentrations of ADT-030.

Supplement 5 **Supplementary Figure 5**. **A.** Western blot analysis showing the expression of LC3 A/B in 2838c3 and MIA PaCa-2 cells treated with DMSO, HCQ, ADT-030, and the combination of HCQ+ADT-030. β-actin was used as a loading control.

Supplement 6 **Supplementary Figure 6**: **A-B.** Serum biochemical analysis of mice treated with ADT-030. Male C57BL/6J mice were treated with vehicle or ADT-030 (150 mg/kg) orally, 5 days/week for 2 weeks. **A.** Serum was collected at the end of the treatment (n=5). Complete blood counts (WBC, RBC, HGB, HCT, MCV, MCH, MCHC, RDW, PLT, MPV, neutrophils, lymphocytes, monocytes, eosinophils, and basophils) revealed no difference between vehicle and ADT-030 treatment. **B.** Biochemical analysis indicated unchanged all measured parameters (total protein, albumin, ALP, ALT, amylase, urea nitrogen, calcium, creatinine, phosphorus, glucose, sodium, potassium, and globulin) except for an increase in total bilirubin because of ADT-030 treatment compared to vehicle treatment. ns: not significant and **p < 0.01.

Supplement 7 **Supplementary Figure 7**: **A.** Open field and locomotor assay revealed no significant different in the overall mobility between vehicle and ADT-030 treated mice (n=5). **B.** ADT-030 plasma concentrations after daily repeated oral administration of 100 mg/kg. **C.** Drug concentrations in lung, kidneys, spleen, heart, liver, brain, ovaries, and colon after oral administration of 100 mg/kg dose. ns: not significant.

Supplement 8 **Supplementary Figure 8**. **A-B.** Tumor images from 2838c3 cell-implanted C57BL/6J mice after treatment with vehicle or ADT-030 at 50 mg/kg (**A**) and 100 mg/kg (**B**).

Supplement 9 **Supplementary Figure 9**. **A.** Percentage of CD45^+^ cells in 2838c3 tumors after treatment with ADT-030 at 150 mg/kg vs. vehicle. **B-H.** Increased percentage of total CD3^+^ T cells (**B**), CD4^+^ T cells (**C**), CD8^+^ T cells (**D**), CD3^+^CTLA4^+^ cells (**E**), CD3^+^PD-1^+^ cells (**F**), CD3^+^LAG3^+^ cells (**G**), CD3^+^TIGIT^+^ cells (**H**) after treatment with ADT-030 vs. vehicle. I. Increased percentage of NK (CD3^−^ NK1.1^+^) cells in vehicle vs. ADT-030 treatment. All quantitative data represent the mean ± SEM. Welch t-test was used for statistical analysis. J. Quantification of RAS-GTP levels in tumor tissues of 2838c-implanted mice treated with either vehicle or 150 mg/kg dose of ADT-030. Welch t-test was used for statistical analysis. ns, non-significant, ∗p < 0.05, ∗∗p < 0.01, ∗∗∗p < 0.001, and ∗∗∗∗p < 0.0001.

Supplement 10 **Supplementary Figure 10**. **A.** Representative Ki-67 IHC results in tumors after vehicle or ADT-030 treatment from the KPC-*f*-luc orthotopic model. **B-D.** Representative IF images of LC3A/B **(B)**, E-Cadherin **(C)**, and vimentin **(D)** in tumor tissues after ADT-030 vs. vehicle treatments. **E.** Bar graph representing the quantification of IHC staining for KI-67. **F-H.** Dot-plot graphs representing the immunofluorescence quantifications of LC3A/B **(F)**, E-cadherin **(G)**, and vimentin **(H)** in tumor tissues after ADT-030 vs. vehicle treatment. Welch t-test was used for statistical analysis. ∗∗pL<L0.01, ∗∗∗pL<L0.001, and ∗∗∗∗pL<L0.0001.

Supplement 11 **Supplementary Figure 11**. ADT-030 modulates tumor immunity in the PDAC TIME in KPC cell-implanted C57BL/6J mice. **A**. Percentage of CD45^+^ immune cells in KPC tumors after vehicle or ADT-030 treatment. **B**. Percentage/mg tumor of total αβ T cells, **C**. γδ (TCRγδ^+^) T cells, **D**. TNK (CD3^+^ NK1.1^+^) cells, **E**. NK (CD3^−^ NK1.1^+^) cells, **F**. NK1.1^+^PD-1^+^ cells **G**. NK1.1^+^CTLA4^+^ cells, **H**. CD4^+^ T cells, **I**. CD4^+^PD-1^+^ cells, CD4^+^TIGIT^+^ cells, CD4^+^CTLA4^+^ cells, CD4^+^ PD-1^+^CTLA4^+^ cells, and CD4^+^FASr^+^ cells, **J**. CD4^+^ T cell subpopulations in tumors from vehicle or ADT-030 treatment. **K**. CD8^+^ T cells, **L**. CD8^+^PD-1^+^ cells, CD8^+^CTLA4^+^ cells, CD8^+^ PD-1^+^CTLA4^+^ cells, CD8^+^LAG3^+^ cells, and CD8^+^ PD-1^+^CTLA4^+^LAG3^+^ cells, **M**. CD8^+^ T cell subpopulations in tumors from vehicle or ADT-030 treatment. **N**. macrophages, **O**. F4/80+ PD-L1+ macrophages, **P**. M1 macrophages, **Q**. M2 macrophages, **R**. M1/M2 ratio in KPC tumors after vehicle or ADT-030 treatment. **S**. Percentage of total dendritic cells/mg tumor, percentage of **T**. cDC1, and **U**. cDC2/mg tumor after ADT-030 vs. vehicle treatment. All quantitative data represents the meanL±LSEM. Welch t-test was used for statistical analysis. ns, non-significant, ∗pL<L0.05, ∗∗pL<L0.01, ∗∗∗pL<L0.001, and ∗∗∗∗pL<L0.0001.

Supplement 12 **Supplementary Figure 12**. **A**. Dot plot showing the markers used to identify various clusters such as PDAC, ADM, macrophages, dendritic cells, T NK & B cells, myocytes, endothelial cells, CAFs, pericytes, and gMDCs. **B**. Dot plot showing the markers used to identify NK cells, T regs, TNK cells, and CD8 T cells. **C**. NK global state trajectory plot displaying pan-activation vs. pan-dysfunction signatures in vehicle and ADT-030 treated tumors. **D**. Dot plot showing expression of activation, dysfunction, and maturation genes in NK cells from vehicle and ADT-030 treated tumors with dot size representing the percentage of expressing cells. **E**. Heat map of differentially expressed genes in NK cells representing a global transcriptional shift toward an activated, cytotoxic program in ADT-030-treated tumors compared to vehicle.

Supplement 13 **Supplementary Figure 13**: **A**-**B**. Ex vivo imaging of livers of KPCML-1-implanted mice treated with vehicle or ADT-030 **(A)** and bar graph representing the bioluminescence quantification **(B)**. **C**-**D**. Ex vivo imaging of lungs in vehicle or ADT-030-treated mice showing reduced metastasis **(C)** and bar graph representing number of distant metastases **(D)**. Welch t-test was used for statistical analysis. pLvalues are listed on bar graphs.

Supplement 14 **Supplementary Figure 14**. **A**. Indicated PDAC cell lines were treated with various concentrations of PF-25465920 for three days followed by determining viable cell number using MTT assays. Relative percentage cell viability was plotted with respect to vehicle (DMSO) treated cells. **B**-**C**. The indicated PDAC cell lines were treated with various concentrations of PF-2545920 for 2–4 weeks, and long-term cell survival was measured using clonogenic assays. Representative images are shown in **(B)** and quantification plotted in **(C)**.

Supplement 15 **Supplementary Figure 15**. **A**. Open field and locomotor assay revealed that mice treated with ADT-030 did not show differences in their mobility compared to vehicle while PF-2545920 produced significant reduction in mobility revealing CNS toxicity. ns, non-significant, ∗pL<L0.05, and ∗∗∗pL<L0.001.

Supplement 16 **Supplementary Figure 16**. The indicated PDAC cell lines were treated with various concentrations of ADT-030 **(A & C)**, MRTX1133 **(B)**, or MRTX849 **(D)** for 2–4 weeks, and long-term cell survival was measured using clonogenic assays.

Supplement 17 **Supplementary Figure 17**. Schematic illustration of the proposed mechanism of action of ADT-030. Inhibition of PDE10 by ADT-030 leads to accumulation of cAMP/cGMP and concomitant activation of PKA/PKG, resulting in both direct antitumor activity and stimulation of antitumor immunity. Direct effects on growth inhibition, induction of apoptosis, and inhibition of metastasis are mediated by suppression of β-catenin/TCF-LEF transcriptional activity and inhibition of both ERK1/2 and PI3K signaling downstream of RAS. Anti-tumor immune effects of ADT-030 are characterized by CD8 T cell-mediated cytotoxicity and immunologic cell death.

Supplement 18

## Data Availability

All data associated with this study are present in the paper or the Supplementary Materials.

## References

[1] Siegel R. L., Giaquinto A. N. & Jemal A. Cancer statistics, 2024. CA: a cancer journal for clinicians 74, 12–49 (2024). 38230766 10.3322/caac.21820

[2] Balsano R., Tommasi C. & Garajova I. State of the art for metastatic pancreatic cancer treatment: Where are we now? Anticancer research 39, 3405–3412 (2019). 31262862 10.21873/anticanres.13484

[3] Eser S., Schnieke A., Schneider G. & Saur D. Oncogenic KRAS signalling in pancreatic cancer. British journal of cancer 111, 817–822 (2014). 24755884 10.1038/bjc.2014.215 PMC4150259

[4] Leonhardt L. & Hebrok M. KRAS degradation averts PDAC chemoresistance. Nature Cancer 5, 375–377 (2024). 38538908 10.1038/s43018-023-00708-7

[5] Zhang Y. β-Catenin mediated TAM phenotype promotes pancreatic cancer metastasis via the OSM/STAT3/LOXL2 axis. Neoplasia 60, 101096 (2025). 39740539 10.1016/j.neo.2024.101096 PMC11745814

[6] Sano M. Activation of WNT/β-catenin signaling enhances pancreatic cancer development and the malignant potential via up-regulation of Cyr61. Neoplasia 18, 785–794 (2016). 27889647 10.1016/j.neo.2016.11.004 PMC5126137

[7] Ram Makena M. Wnt/β-catenin signaling: the culprit in pancreatic carcinogenesis and therapeutic resistance. International journal of molecular sciences 20, 4242 (2019). 31480221 10.3390/ijms20174242 PMC6747343

[8] Manegold P. Differentiation therapy targeting the β-catenin/CBP interaction in pancreatic cancer. Cancers 10, 95 (2018). 29596326 10.3390/cancers10040095 PMC5923350

[9] Dominguez A. A. Unveiling the Promise: Navigating Clinical Trials 1978–2024 for PDAC. Cancers 16, 3564 (2024). 39518005 10.3390/cancers16213564 PMC11544830

[10] Jiménez D. J., Javed A., Rubio-Tomás T., Seye-Loum N. & Barceló C. Clinical and preclinical targeting of oncogenic pathways in PDAC: targeted therapeutic approaches for the deadliest cancer. International journal of molecular sciences 25, 2860 (2024). 38474109 10.3390/ijms25052860 PMC10932149

[11] Huber M. The immune microenvironment in pancreatic cancer. International journal of molecular sciences 21, 7307 (2020). 33022971 10.3390/ijms21197307 PMC7583843

[12] Yousef A. Impact of KRAS mutations and co-mutations on clinical outcomes in pancreatic ductal adenocarcinoma. NPJ Precision Oncology 8, 27 (2024). 38310130 10.1038/s41698-024-00505-0 PMC10838312

[13] Zhang Z., Zhang H., Liao X. & Tsai H.-i. KRAS mutation: The booster of pancreatic ductal adenocarcinoma transformation and progression. Frontiers in Cell and Developmental Biology 11, 1147676 (2023). 37152291 10.3389/fcell.2023.1147676 PMC10157181

[14] Li J., Mizukami Y., Zhang X., Jo W.-S. & Chung D. C. Oncogenic K-ras stimulates Wnt signaling in colon cancer through inhibition of GSK-3β. Gastroenterology 128, 1907–1918 (2005). 15940626 10.1053/j.gastro.2005.02.067

[15] Shetu S. A. & Bandyopadhyay D. Small-molecule RAS inhibitors as anticancer agents: discovery, development, and mechanistic studies. International Journal of Molecular Sciences 23, 3706 (2022). 35409064 10.3390/ijms23073706 PMC8999084

[16] Ryu W.-J., Han G., Lee S.-H. & Choi K.-Y. Suppression of Wnt/β-catenin and RAS/ERK pathways provides a therapeutic strategy for gemcitabine-resistant pancreatic cancer. Biochemical and Biophysical Research Communications 549, 40–46 (2021). 33662667 10.1016/j.bbrc.2021.02.076

[17] Wang W. Dual-targeted therapy circumvents non-genetic drug resistance to targeted therapy. Frontiers in Oncology 12, 859455 (2022). 35574302 10.3389/fonc.2022.859455 PMC9093074

[18] Raghavendra N. M., Pingili D., Kadasi S., Mettu A. & Prasad S. Dual or multi-targeting inhibitors: The next generation anticancer agents. European journal of medicinal chemistry 143, 1277–1300 (2018). 29126724 10.1016/j.ejmech.2017.10.021

[19] Lemieux E., Cagnol S., Beaudry K., Carrier J. & Rivard N. Oncogenic KRAS signalling promotes the Wnt/β-catenin pathway through LRP6 in colorectal cancer. Oncogene 34, 4914–4927 (2015). 25500543 10.1038/onc.2014.416 PMC4687460

[20] Samidurai A. Role of phosphodiesterase 1 in the pathophysiology of diseases and potential therapeutic opportunities. Pharmacology & therapeutics 226, 107858 (2021). 33895190 10.1016/j.pharmthera.2021.107858 PMC8815087

[21] Gross N. E. Phosphodiesterase-5 inhibition collaborates with vaccine-based immunotherapy to reprogram myeloid cells in pancreatic ductal adenocarcinoma. JCI insight 9, e179292 (2024). 39106104 10.1172/jci.insight.179292 PMC11457845

[22] Kelly M. P. Cyclic nucleotide phosphodiesterases as drug targets. Pharmacological reviews 77 (2025). 10.1016/j.pharmr.2025.100042 PMC13095499 40081105

[23] Faure M. & Bourne H. Differential effects on cAMP on the MAP kinase cascade: evidence for a cAMP-insensitive step that can bypass Raf-1. Molecular biology of the cell 6, 1025–1035 (1995). 7579705 10.1091/mbc.6.8.1025 PMC301260

[24] Suhasini M., Li H., Lohmann S. M., Boss G. R. & Pilz R. B. Cyclic-GMP-dependent protein kinase inhibits the Ras/Mitogen-activated protein kinase pathway. Molecular and cellular biology 18, 6983–6994 (1998). 9819386 10.1128/mcb.18.12.6983 PMC109281

[25] Borneman R. M. Phosphodiesterase 10A (PDE10A) as a novel target to suppress β-catenin and RAS signaling in epithelial ovarian cancer. Journal of Ovarian Research 15, 120 (2022). 36324187 10.1186/s13048-022-01050-9 PMC9632086

[26] Jeong W.-J., Ro E. J. & Choi K.-Y. Interaction between Wnt/β-catenin and RAS-ERK pathways and an anti-cancer strategy via degradations of β-catenin and RAS by targeting the Wnt/β-catenin pathway. NPJ Precision oncology 2, 5 (2018). 29872723 10.1038/s41698-018-0049-y PMC5871897

[27] Zhu B. Phosphodiesterase 10A is overexpressed in lung tumor cells and inhibitors selectively suppress growth by blocking β-catenin and MAPK signaling. Oncotarget 8, 69264 (2017). 29050202 10.18632/oncotarget.20566 PMC5642477

[28] Peng T. Inhibitors of phosphodiesterase as cancer therapeutics. European journal of medicinal chemistry 150, 742–756 (2018). 29574203 10.1016/j.ejmech.2018.03.046

[29] Li N. Phosphodiesterase 10A: a novel target for selective inhibition of colon tumor cell growth and β-catenin-dependent TCF transcriptional activity. Oncogene 34, 1499–1509 (2015). 24704829 10.1038/onc.2014.94 PMC4212019

[30] Lee K. J. Suppression of colon tumorigenesis in mutant apc mice by a novel PDE10 inhibitor that reduces oncogenic β-catenin. Cancer Prevention Research 14, 995–1008 (2021). 34584001 10.1158/1940-6207.CAPR-21-0208 PMC8916418

[31] Loveless I. M. Human Pancreatic Cancer Single-Cell Atlas Reveals Association of CXCL10+ Fibroblasts and Basal Subtype Tumor Cells. Clinical cancer research 31, 756–772 (2025). 39636224 10.1158/1078-0432.CCR-24-2183 PMC11831110

[32] Babic I. MICRO-TAG enzyme complementation enables quantification of cellular drug-target engagement in temperature series. SLAS Discovery, 100291 (2025). 41285198 10.1016/j.slasd.2025.100291

[33] Tinsley H. N. Sulindac sulfide selectively inhibits growth and induces apoptosis of human breast tumor cells by phosphodiesterase 5 inhibition, elevation of cyclic GMP, and activation of protein kinase G. Molecular cancer therapeutics 8, 3331–3340 (2009). 19996273 10.1158/1535-7163.MCT-09-0758 PMC2805153

[34] Kim D.-Y., Park J.-S., Leem Y.-H., Park J.-E. & Kim H.-S. The potent PDE10A inhibitor MP-10 (PF-2545920) suppresses microglial activation in LPS-induced neuroinflammation and MPTP-induced Parkinson’s disease mouse models. Journal of Neuroimmune Pharmacology 16, 470–482 (2021). 32671618 10.1007/s11481-020-09943-6

[35] Seibenhener M. L. & Wooten M. C. Use of the open field maze to measure locomotor and anxiety-like behavior in mice. Journal of visualized experiments: JoVE, 52434 (2015). 25742564 10.3791/52434 PMC4354627

[36] Bandi D. S. R. ADT-1004: A promising pan-RAS inhibitor for targeting KRAS mutations in pancreatic ductal adenocarcinoma. Cancer Research 84, 5915–5915 (2024).

[37] Reinhard M. The 46/50 kDa phosphoprotein VASP purified from human platelets is a novel protein associated with actin filaments and focal contacts. The EMBO journal 11, 2063–2070 (1992). 1318192 10.1002/j.1460-2075.1992.tb05264.x PMC556672

[38] Üffing A., Attridge E. & Tooze S. A. Targeting an alternative route: autophagy in RAS-driven cancers. Cell Research 35, 389–390 (2025). 40374948 10.1038/s41422-025-01127-2 PMC12134140

[39] Gurpinar E. A novel sulindac derivative inhibits lung adenocarcinoma cell growth through suppression of Akt/mTOR signaling and induction of autophagy. Molecular cancer therapeutics 12, 663–674 (2013). 23443799 10.1158/1535-7163.MCT-12-0785 PMC3651802

[40] Berven L. A. & Crouch M. F. Cellular function of p70S6K: a role in regulating cell motility. Immunology and cell biology 78, 447–451 (2000). 10947872 10.1046/j.1440-1711.2000.00928.x

[41] Tu Z. Radiosynthesis and in vivo evaluation of [11C] MP-10 as a PET probe for imaging PDE10A in rodent and non-human primate brain. Bioorganic & medicinal chemistry 19, 1666–1673 (2011). 21315609 10.1016/j.bmc.2011.01.032 PMC3056285

[42] Spadafora V. Optimization of a mouse model of pancreatic cancer to simulate the human phenotypes of metastasis and cachexia. BMC cancer 24, 414 (2024). 38570770 10.1186/s12885-024-12104-0 PMC10993462

[43] Hallin J. Anti-tumor efficacy of a potent and selective non-covalent KRASG12D inhibitor. Nature medicine 28, 2171–2182 (2022). 10.1038/s41591-022-02007-7 36216931

[44] Hallin J. The KRASG12C inhibitor MRTX849 provides insight toward therapeutic susceptibility of KRAS-mutant cancers in mouse models and patients. Cancer discovery 10, 54–71 (2020). 31658955 10.1158/2159-8290.CD-19-1167 PMC6954325

[45] Bahar M. E., Kim H. J. & Kim D. R. Targeting the RAS/RAF/MAPK pathway for cancer therapy: from mechanism to clinical studies. Signal transduction and targeted therapy 8, 455 (2023). 38105263 10.1038/s41392-023-01705-z PMC10725898

[46] Choucair K. Targeting KRAS mutations: orchestrating cancer evolution and therapeutic challenges. Signal Transduction and Targeted Therapy 10, 385 (2025). 41309533 10.1038/s41392-025-02473-8 PMC12660860

[47] Yu F. Wnt/β-catenin signaling in cancers and targeted therapies. Signal Transduction and Targeted Therapy 6, 307 (2021). 34456337 10.1038/s41392-021-00701-5 PMC8403677

[48] Xu H., Ren S., Wang Y., Zhang T. & Lu J. Abnormal activation of the Ras/MAPK signaling pathway in oncogenesis and progression. Cancer Adv 8, e25002 (2025).

[49] Kim E. Promotion of growth factor signaling as a critical function of β-catenin during HCC progression. Nature communications 10, 1909 (2019). 10.1038/s41467-019-09780-z PMC6478918 31015417

[50] Zeng G. Aberrant Wnt/β-catenin signaling in pancreatic adenocarcinoma. Neoplasia 8, 279–289 (2006). 16756720 10.1593/neo.05607 PMC1600679

[51] Lee S.-K., Hwang J.-H. & Choi K.-Y. Interaction of the Wnt/β-catenin and RAS-ERK pathways involving co-stabilization of both β-catenin and RAS plays important roles in the colorectal tumorigenesis. Advances in biological regulation 68, 46–54 (2018). 29449169 10.1016/j.jbior.2018.01.001

[52] Dumaz N. & Marais R. Protein kinase A blocks Raf-1 activity by stimulating 14–3-3 binding and blocking Raf-1 interaction with Ras. Journal of Biological Chemistry 278, 29819–29823 (2003). 12801936 10.1074/jbc.C300182200

[53] Bye B. A. Combined PI3K and MAPK inhibition synergizes to suppress PDAC. bioRxiv, 2023.2008. 2015.553438 (2023).

[54] Ouissam A. J., Hind C., Sami Aziz B. & Said A. Inhibition of the PI3K/AKT/mTOR pathway in pancreatic cancer: is it a worthwhile endeavor? Therapeutic advances in medical oncology 16, 17588359241284911 (2024). 10.1177/17588359241284911 PMC11468005 39399412

[55] Awasthi N. Dual inhibition of the PI3K and MAPK pathways enhances nab-paclitaxel/gemcitabine chemotherapy response in preclinical models of pancreatic cancer. Cancer Letters 459, 41–49 (2019). 31153980 10.1016/j.canlet.2019.05.037

[56] Uthayathas S. Phosphodiesterase 10A inhibitor MP-10 effects in primates: comparison with risperidone and mechanistic implications. Neuropharmacology 77, 257–267 (2014). 24490227 10.1016/j.neuropharm.2013.10.015 PMC3934827

[57] Ju Y. Barriers and opportunities in pancreatic cancer immunotherapy. NPJ Precision Oncology 8, 199 (2024). 39266715 10.1038/s41698-024-00681-z PMC11393360

[58] Ye N. A multi-omic approach reveals utility of CD45 expression in prognosis and novel target discovery. Frontiers in Genetics 13, 928328 (2022). 36061172 10.3389/fgene.2022.928328 PMC9428580

[59] Pollini T. The tumour immune microenvironment and microbiome of pancreatic intraductal papillary mucinous neoplasms. The Lancet Gastroenterology & Hepatology 7, 1141–1150 (2022). 36057265 10.1016/S2468-1253(22)00235-7 PMC9844533

[60] Kabacaoglu D., Ciecielski K. J., Ruess D. A. & Algül H. Immune checkpoint inhibition for pancreatic ductal adenocarcinoma: current limitations and future options. Frontiers in immunology 9, 1878 (2018). 30158932 10.3389/fimmu.2018.01878 PMC6104627

[61] Wang L. PD-L1-expressing tumor-associated macrophages are immunostimulatory and associate with good clinical outcome in human breast cancer. Cell Reports Medicine 5 (2024). 10.1016/j.xcrm.2024.101420 PMC10897617 38382468

[62] Böttcher J. P. NK cells stimulate recruitment of cDC1 into the tumor microenvironment promoting cancer immune control. Cell 172, 1022–1037. e1014 (2018). 29429633 10.1016/j.cell.2018.01.004 PMC5847168

[63] Tanaka N. Clinical acquired resistance to KRASG12C inhibition through a novel KRAS switch-II pocket mutation and polyclonal alterations converging on RAS–MAPK reactivation. Cancer discovery 11, 1913–1922 (2021). 33824136 10.1158/2159-8290.CD-21-0365 PMC8338755

[64] Li Y., Zhao J. & Li Y. New exploration of KRASG12D inhibitors and the mechanisms of resistance. Experimental Hematology & Oncology 14, 39 (2025). 40097966 10.1186/s40164-025-00637-4 PMC11912584

[65] Awad M. M. Acquired resistance to KRASG12C inhibition in cancer. New England Journal of Medicine 384, 2382–2393 (2021). 34161704 10.1056/NEJMoa2105281 PMC8864540

