# Supplementary figures and images for: ADT-030, a novel PDE10 inhibitor, demonstrates potent antitumor activity in pancreatic ductal adenocarcinoma
Source: bioRxiv. 2026 Feb 13:2026.02.11.705411. Preprint. [Version 1] doi: 10.64898/2026.02.11.705411 (PMC12918786; doi:10.64898/2026.02.11.705411)

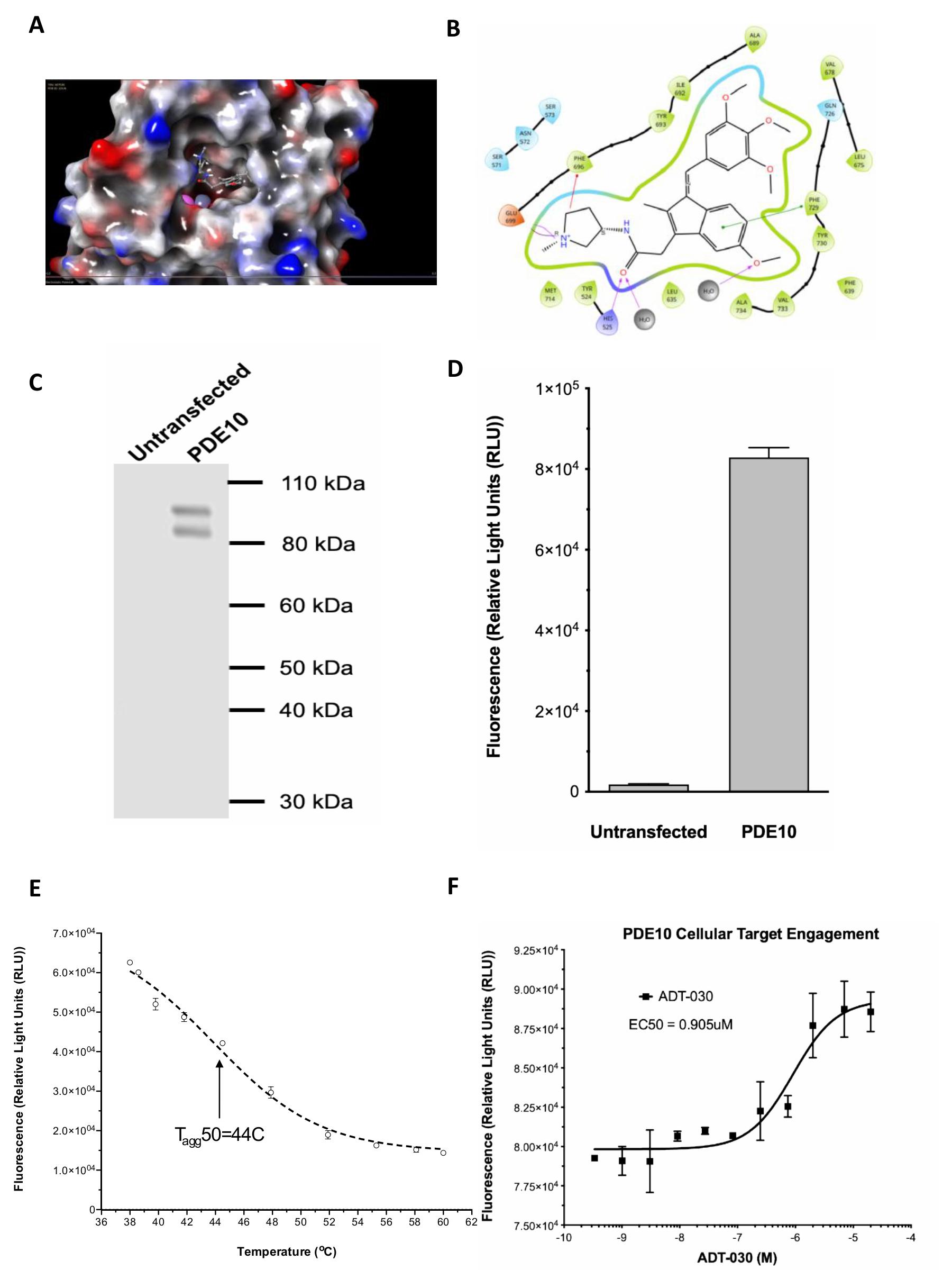

Supplement: Supplement 1 — Supplementary Figure 1: A. Docking of ADT-030 to PDE10 (2OUN) resulted in an optimal docking score of −10.325. Representative surface rendering of 2OUN with electrostatic potential mapped onto the surface shows ADT-030 bound in the PDE10 catalytic pocket. ADT-030 is rendered as a ball-and-stick representation. Pink and grey spheres illustrate magnesium and zinc ions in the pocket, respectively. B. Interaction diagram showing molecular interactions of ADT-030 with PDE10. Binding affinity of ADT-030 to PDE10 as determined by treating HEK293 cells (45 min) expressing PDE10-Micro-Tag with ADT-030. C. Western blot analysis showing PDE10 expression in HEK293 cells transfected with PDE10-Micro-Tag and detected using anti-Micro-Tag antibody. D. Quantification of Micro-Tag enzyme complementation in cells transfected with PDE10-Micro-Tag construct vs untransfected HEK293 cells. E. PDE10 thermal curve yielding a Tagg50 of 44°C, providing the fixed challenge temperature to determine ADT-030 binding to PDE10 in transfected HEK293 cells. F. ADT-030 binding to PDE10 in transfected HEK293 cells. The curve is graphed as the average of two replicates ± SEM. [file media-1.jpg]

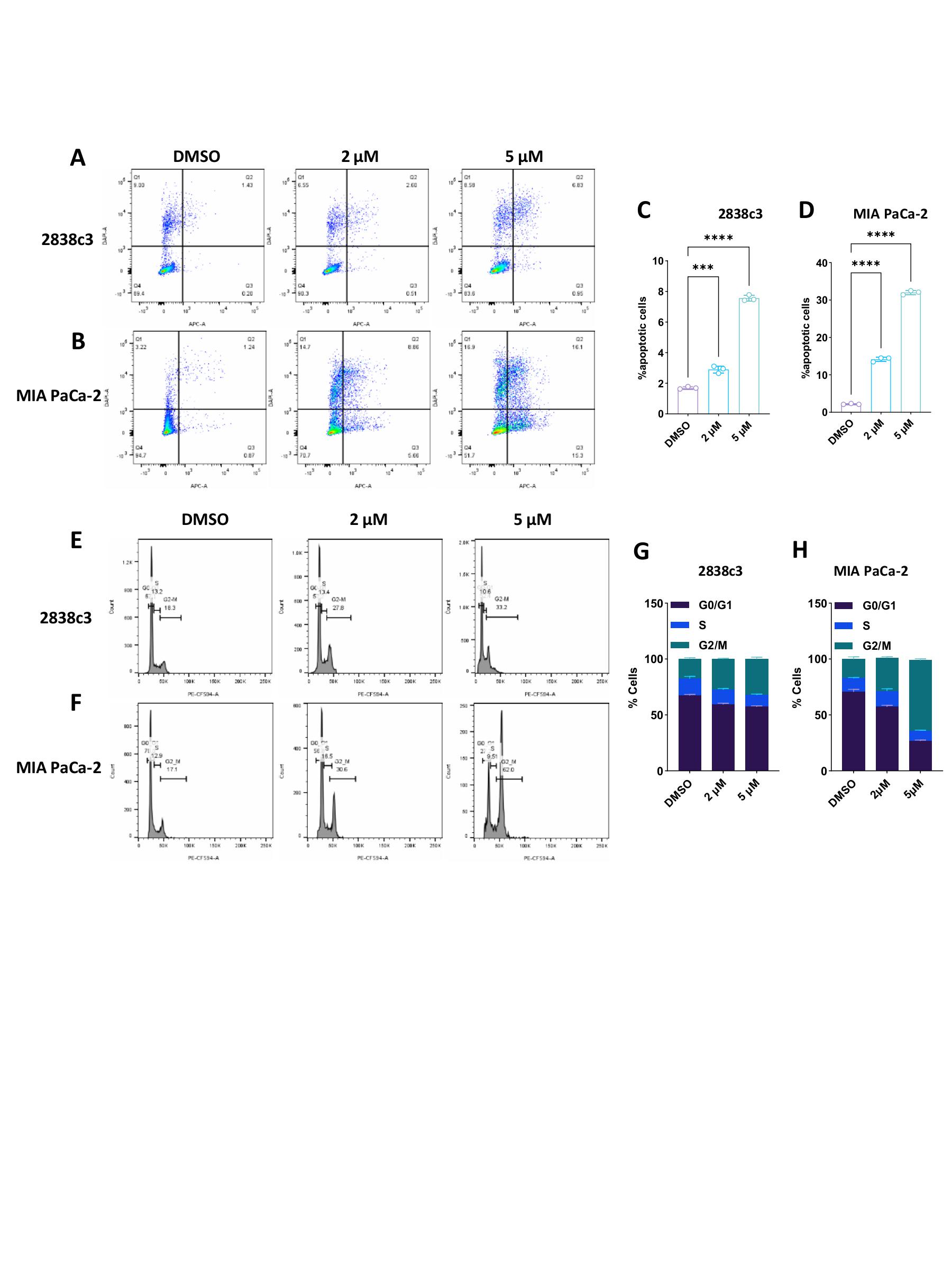

Supplement: Supplement 2 — Supplementary Figure 2: A-B. Representative FACS analysis showing ADT-030 induced apoptosis in 2838c3 (A), and MIA PaCa-2 (B) cells after treatment with ADT-030 (2 and 5 μM) or vehicle for 24 hrs. After double-staining with annexin V and PI, cells were subjected to flow cytometry analysis. C-D. The indicated PDAC cell lines were treated with ADT-030 at varying concentrations for 24 hrs, and apoptosis was measured following annexin V/propidium iodide labeling. E-F. Representative DNA histogram showing cell cycle arrest in 2838c3 (E), and MIA PaCa-2 (F) cells after treatment with the indicated concentrations of ADT-030 or vehicle controls for 72 hrs. G-H. Flow cytometry analysis of cell cycle distribution in 2838c3 (G) and MIA PaCa-2 (H) cells treated with vehicle or ADT-030 at the indicated concentrations. Data represents the mean ± SEMr of three biological replicates. ns = not significant, *p < 0.05, **p < 0.01, ***p < 0.001, ****p < 0.0001. (one-way ANOVA). [file media-2.jpg]

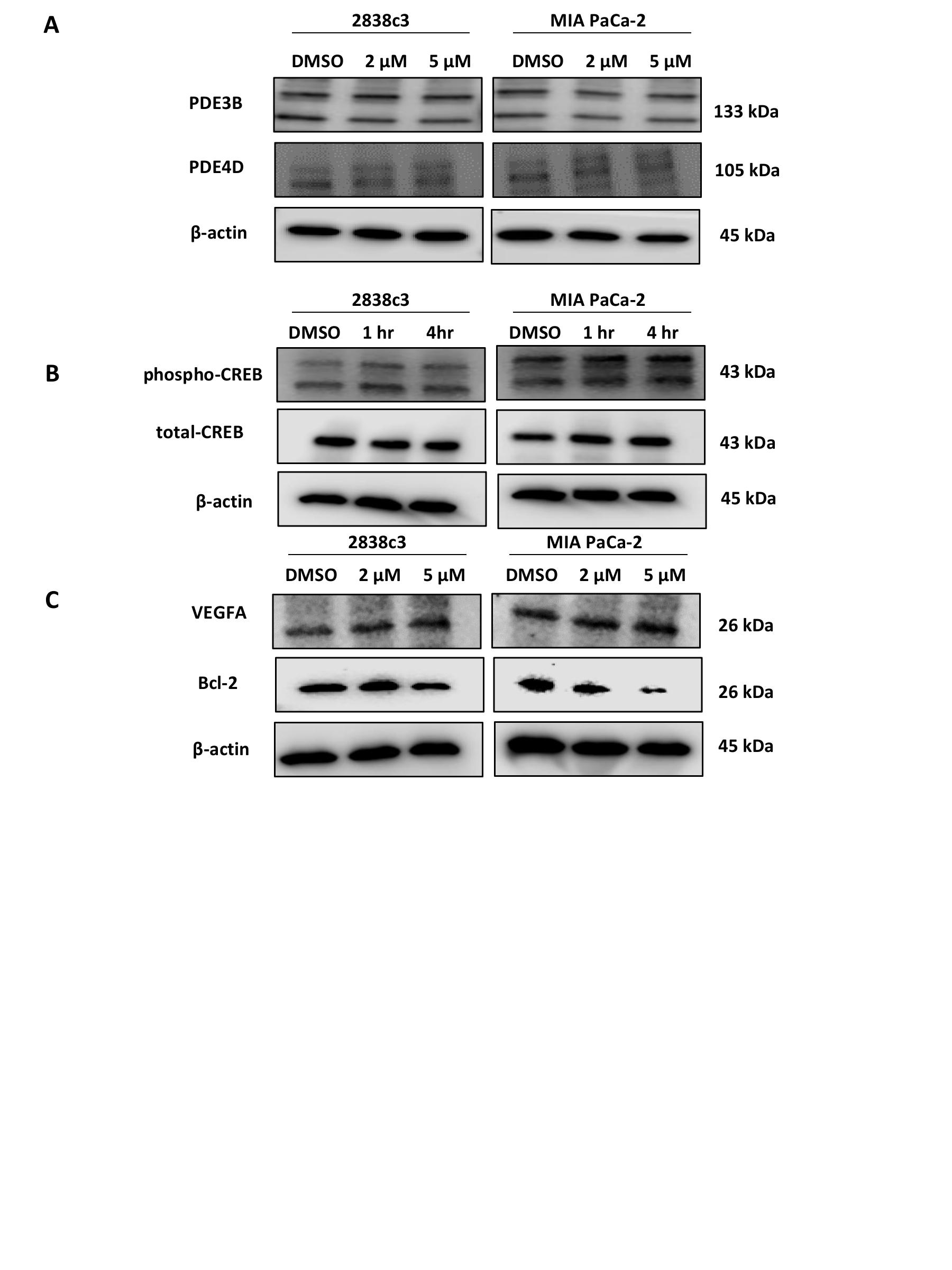

Supplement: Supplement 3 — Supplementary Figure 3: A-C. Western blot analysis showing the expression of indicated PDE isozymes, pCREB CREB, VEGFA, and Bcl-2 in 2838c3 and MIA PaCa-2 cells treated with DMSO, and varied time points/concentrations of ADT-030. [file media-3.jpg]

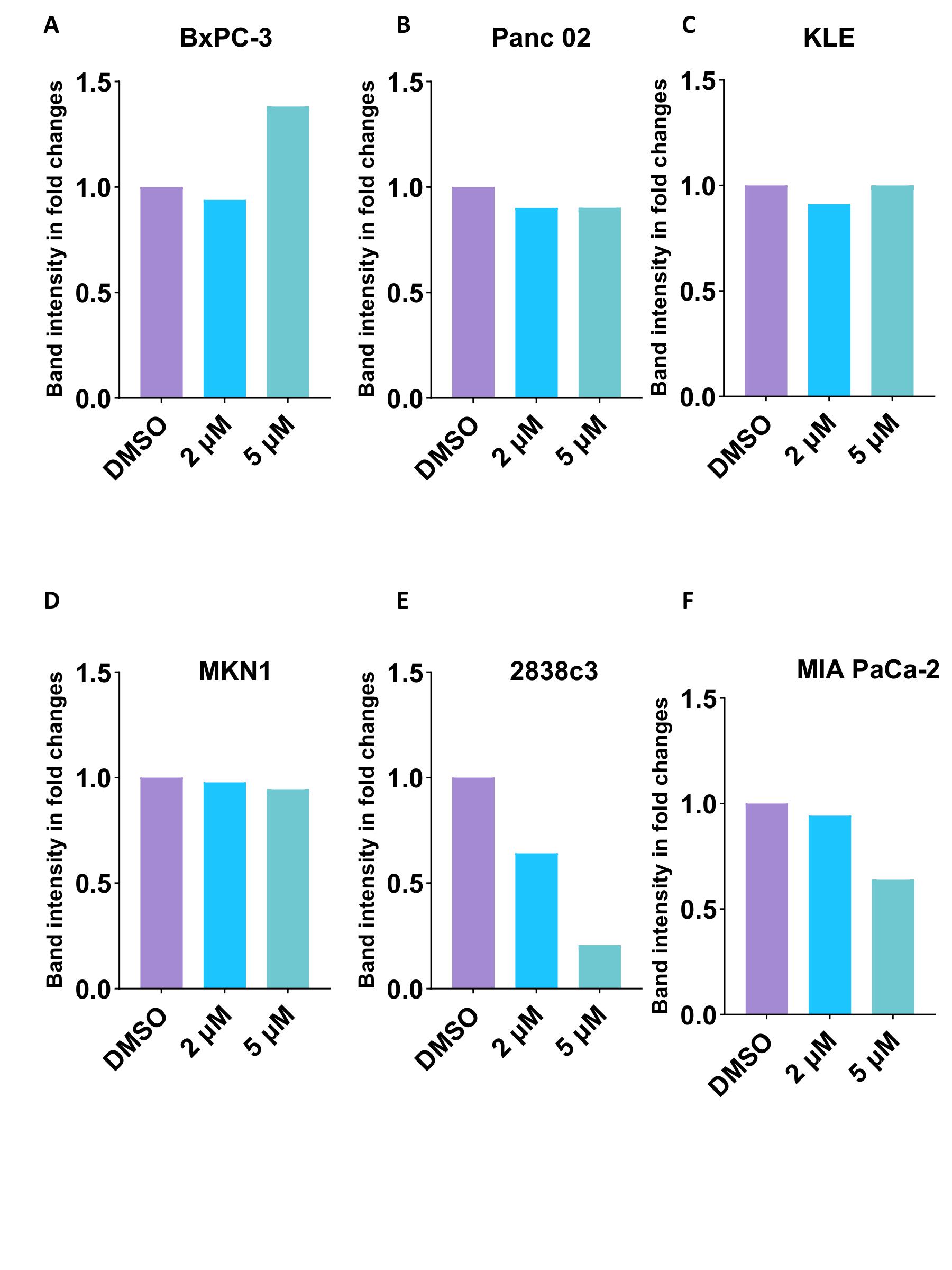

Supplement: Supplement 4 — Supplementary Figure 4: A-F. Quantification of RAS-GTP activation in BxPC-3, Panc 02, KLE, MKN1, 2838c3, and MIAPaCa-2 cells treated with either DMSO or increasing concentrations of ADT-030. [file media-4.jpg]

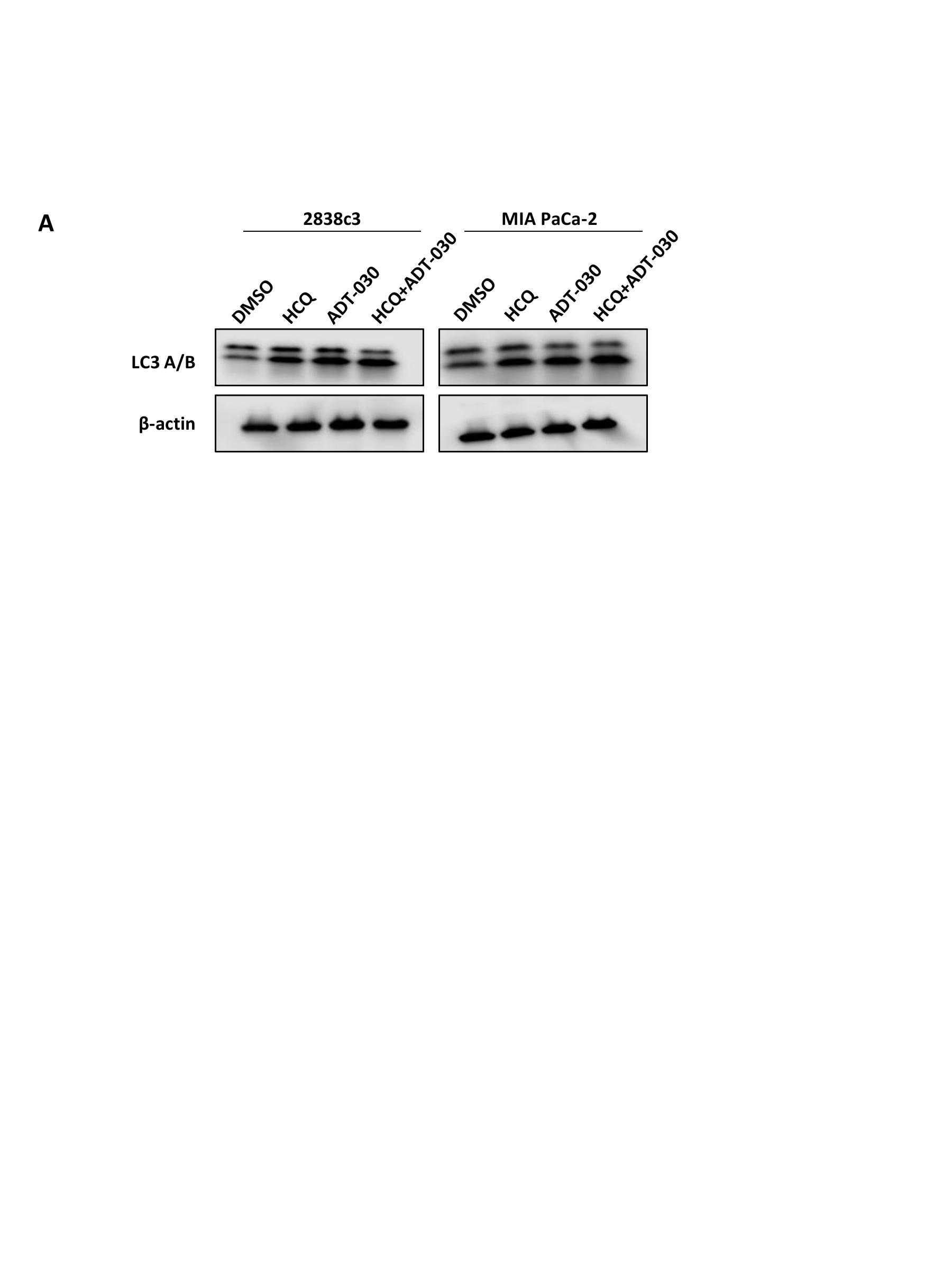

Supplement: Supplement 5 — Supplementary Figure 5. A. Western blot analysis showing the expression of LC3 A/B in 2838c3 and MIA PaCa-2 cells treated with DMSO, HCQ, ADT-030, and the combination of HCQ+ADT-030. β-actin was used as a loading control. [file media-5.jpg]

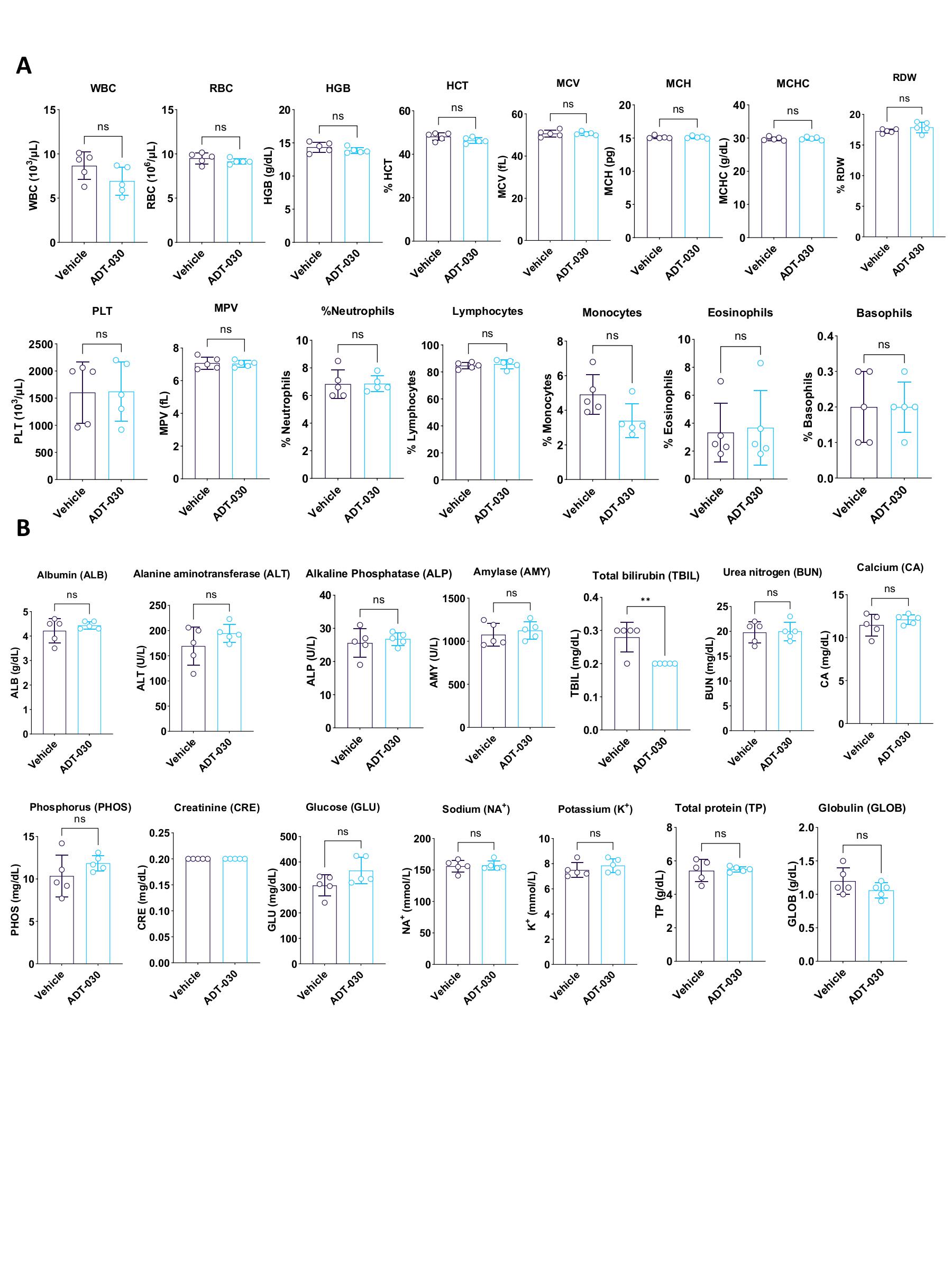

Supplement: Supplement 6 — Supplementary Figure 6: A-B. Serum biochemical analysis of mice treated with ADT-030. Male C57BL/6J mice were treated with vehicle or ADT-030 (150 mg/kg) orally, 5 days/week for 2 weeks. A. Serum was collected at the end of the treatment (n=5). Complete blood counts (WBC, RBC, HGB, HCT, MCV, MCH, MCHC, RDW, PLT, MPV, neutrophils, lymphocytes, monocytes, eosinophils, and basophils) revealed no difference between vehicle and ADT-030 treatment. B. Biochemical analysis indicated unchanged all measured parameters (total protein, albumin, ALP, ALT, amylase, urea nitrogen, calcium, creatinine, phosphorus, glucose, sodium, potassium, and globulin) except for an increase in total bilirubin because of ADT-030 treatment compared to vehicle treatment. ns: not significant and **p < 0.01. [file media-6.jpg]

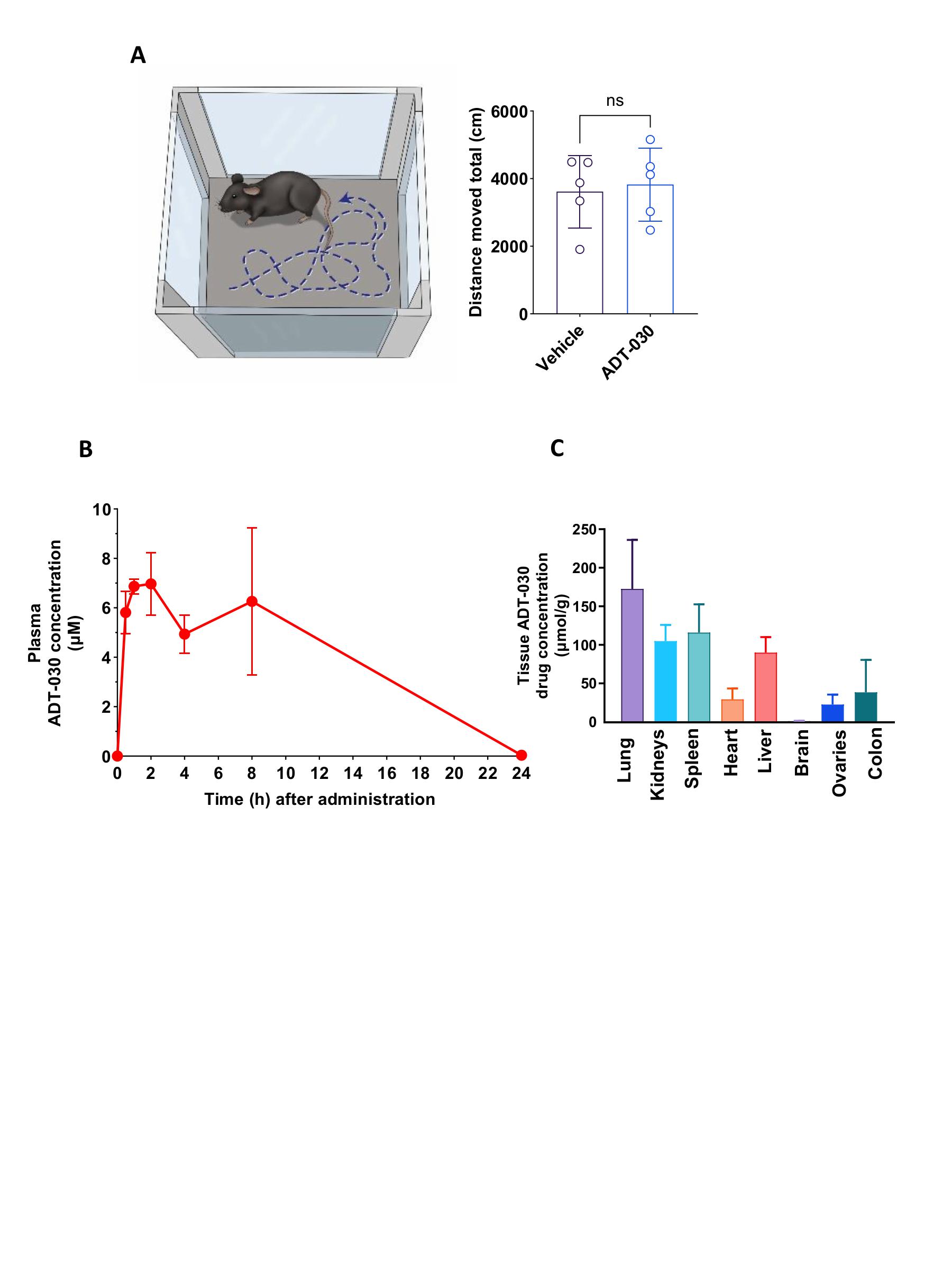

Supplement: Supplement 7 — Supplementary Figure 7: A. Open field and locomotor assay revealed no significant different in the overall mobility between vehicle and ADT-030 treated mice (n=5). B. ADT-030 plasma concentrations after daily repeated oral administration of 100 mg/kg. C. Drug concentrations in lung, kidneys, spleen, heart, liver, brain, ovaries, and colon after oral administration of 100 mg/kg dose. ns: not significant. [file media-7.jpg]

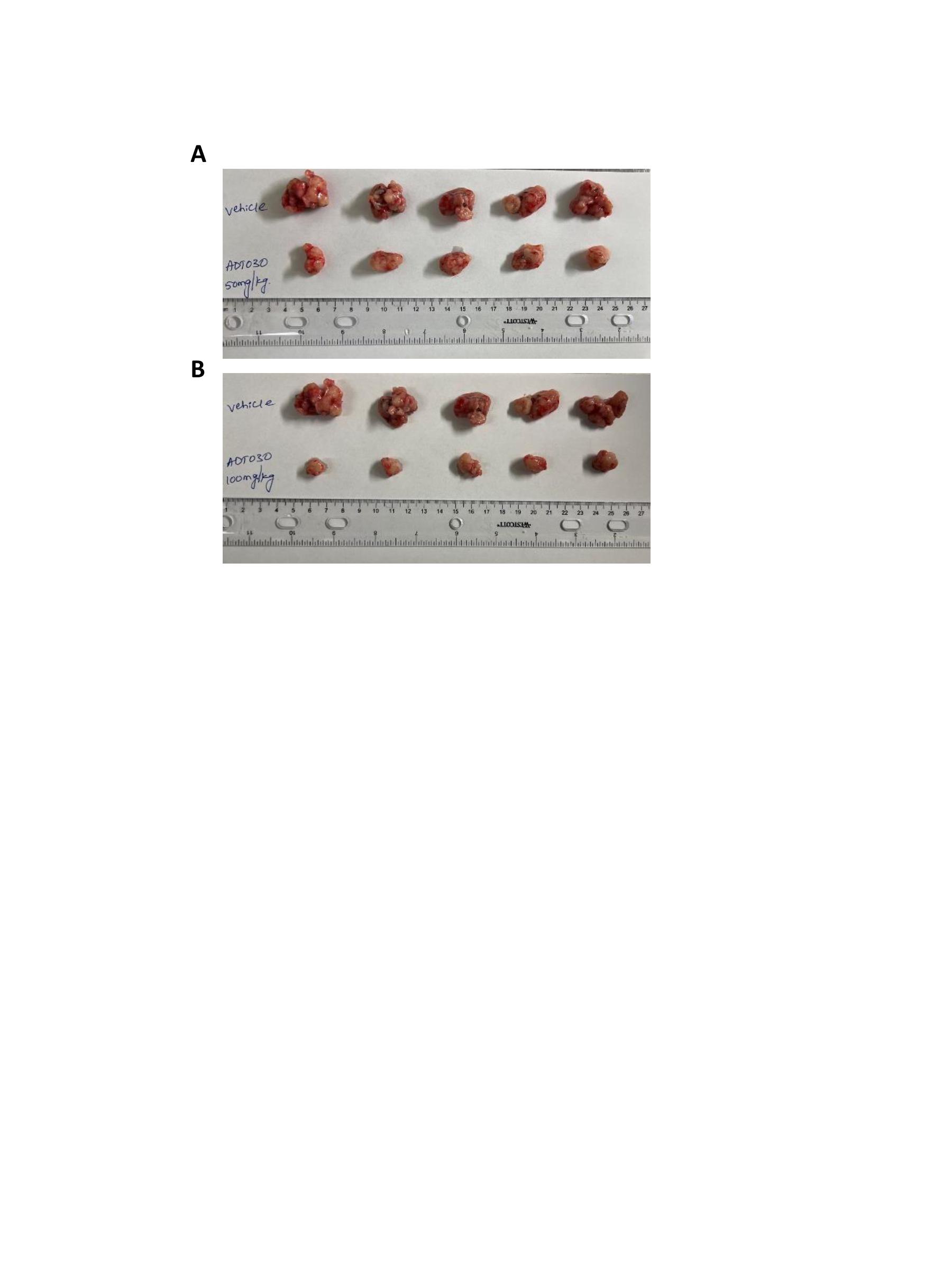

Supplement: Supplement 8 — Supplementary Figure 8. A-B. Tumor images from 2838c3 cell-implanted C57BL/6J mice after treatment with vehicle or ADT-030 at 50 mg/kg (A) and 100 mg/kg (B). [file media-8.jpg]

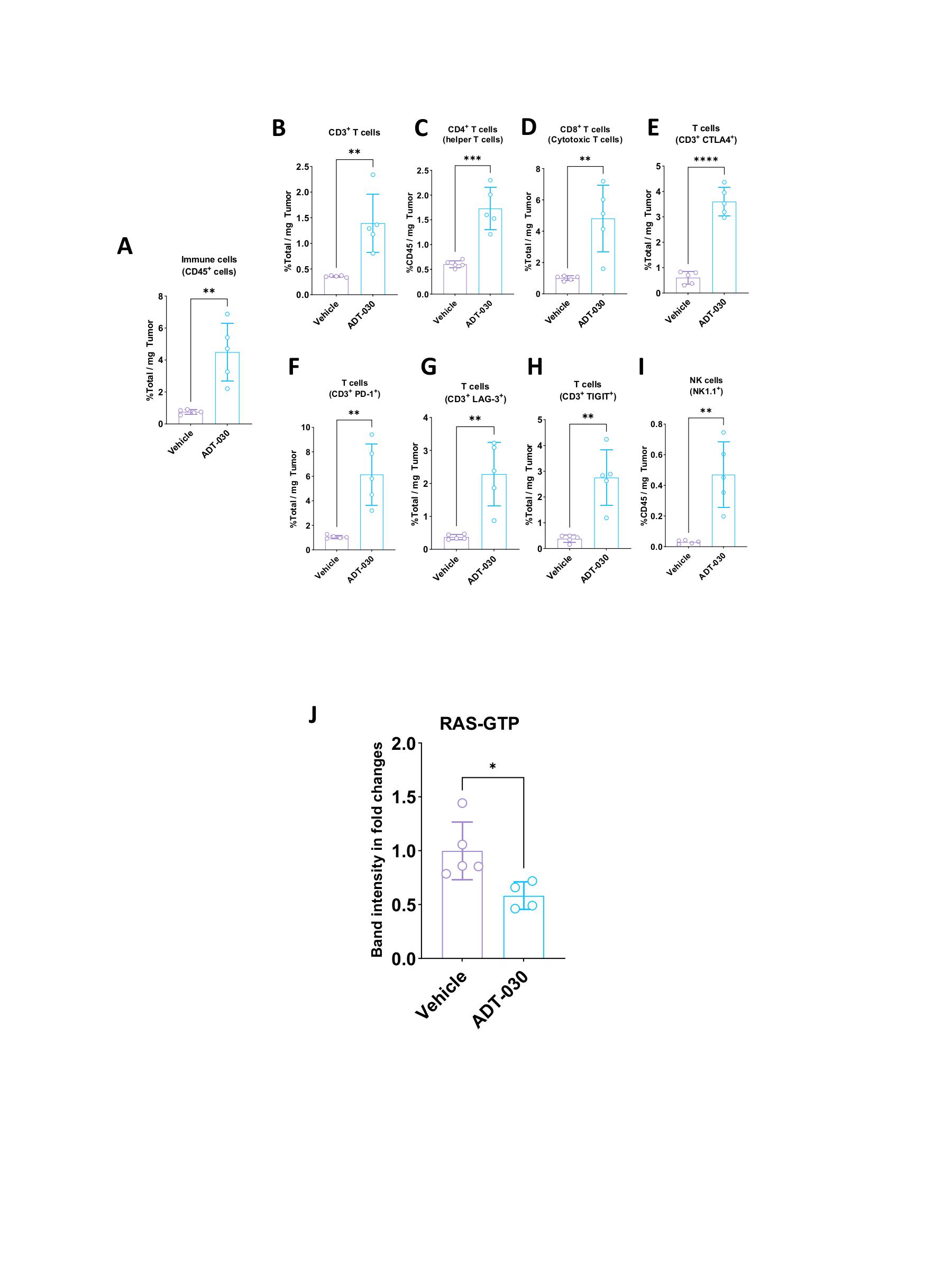

Supplement: Supplement 9 — Supplementary Figure 9. A. Percentage of CD45+ cells in 2838c3 tumors after treatment with ADT-030 at 150 mg/kg vs. vehicle. B-H. Increased percentage of total CD3+ T cells (B), CD4+ T cells (C), CD8+ T cells (D), CD3+CTLA4+ cells (E), CD3+PD-1+ cells (F), CD3+LAG3+ cells (G), CD3+TIGIT+ cells (H) after treatment with ADT-030 vs. vehicle. I. Increased percentage of NK (CD3− NK1.1+) cells in vehicle vs. ADT-030 treatment. All quantitative data represent the mean ± SEM. Welch t-test was used for statistical analysis. J. Quantification of RAS-GTP levels in tumor tissues of 2838c-implanted mice treated with either vehicle or 150 mg/kg dose of ADT-030. Welch t-test was used for statistical analysis. ns, non-significant, ∗p < 0.05, ∗∗p < 0.01, ∗∗∗p < 0.001, and ∗∗∗∗p < 0.0001. [file media-9.jpg]

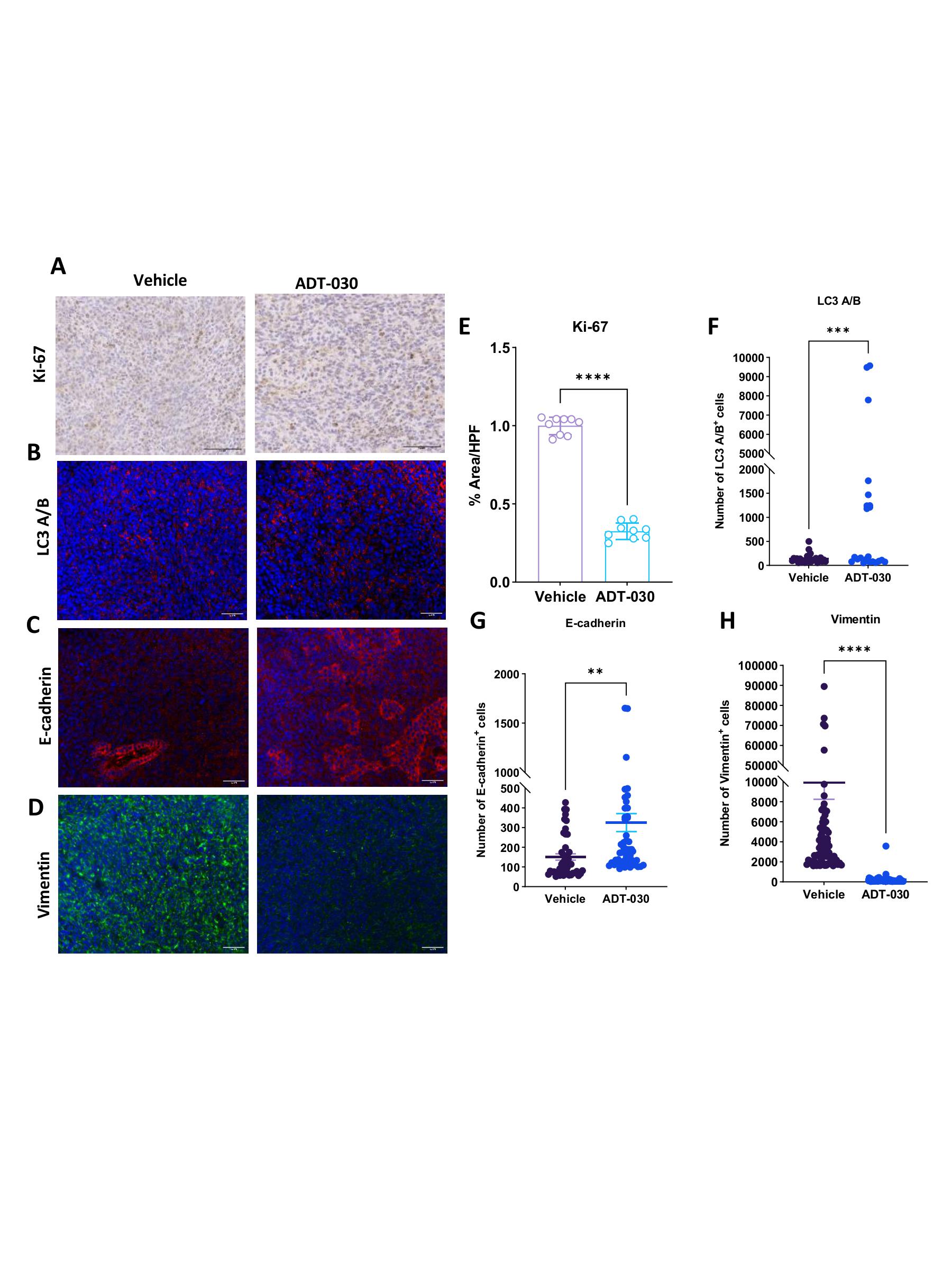

Supplement: Supplement 10 — Supplementary Figure 10. A. Representative Ki-67 IHC results in tumors after vehicle or ADT-030 treatment from the KPC-f-luc orthotopic model. B-D. Representative IF images of LC3A/B (B), E-Cadherin (C), and vimentin (D) in tumor tissues after ADT-030 vs. vehicle treatments. E. Bar graph representing the quantification of IHC staining for KI-67. F-H. Dot-plot graphs representing the immunofluorescence quantifications of LC3A/B (F), E-cadherin (G), and vimentin (H) in tumor tissues after ADT-030 vs. vehicle treatment. Welch t-test was used for statistical analysis. ∗∗pL<L0.01, ∗∗∗pL<L0.001, and ∗∗∗∗pL<L0.0001. [file media-10.jpg]

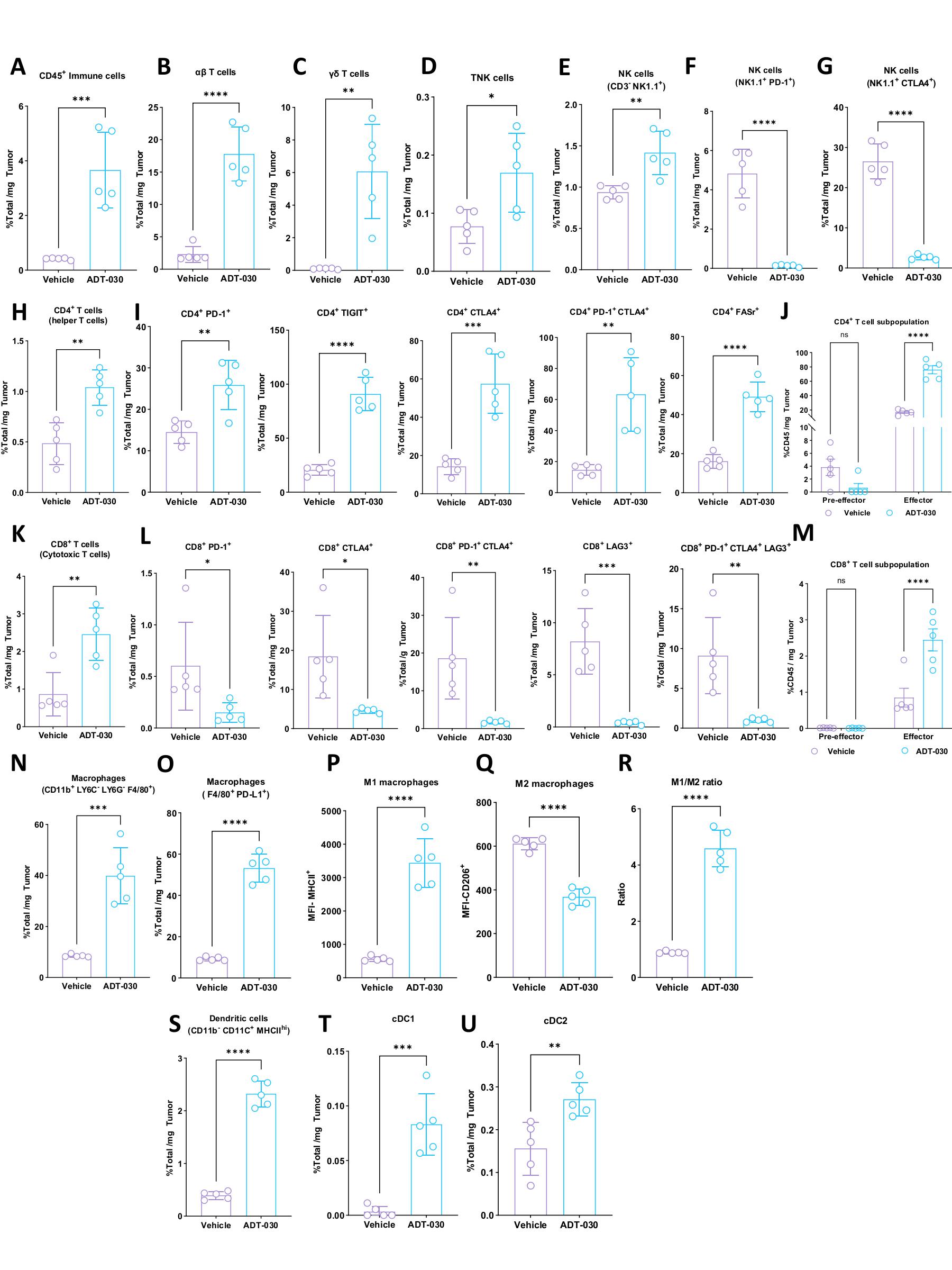

Supplement: Supplement 11 — Supplementary Figure 11. ADT-030 modulates tumor immunity in the PDAC TIME in KPC cell-implanted C57BL/6J mice. A. Percentage of CD45+ immune cells in KPC tumors after vehicle or ADT-030 treatment. B. Percentage/mg tumor of total αβ T cells, C. γδ (TCRγδ+) T cells, D. TNK (CD3+ NK1.1+) cells, E. NK (CD3− NK1.1+) cells, F. NK1.1+PD-1+ cells G. NK1.1+CTLA4+ cells, H. CD4+ T cells, I. CD4+PD-1+ cells, CD4+TIGIT+ cells, CD4+CTLA4+ cells, CD4+ PD-1+CTLA4+ cells, and CD4+FASr+ cells, J. CD4+ T cell subpopulations in tumors from vehicle or ADT-030 treatment. K. CD8+ T cells, L. CD8+PD-1+ cells, CD8+CTLA4+ cells, CD8+ PD-1+CTLA4+ cells, CD8+LAG3+ cells, and CD8+ PD-1+CTLA4+LAG3+ cells, M. CD8+ T cell subpopulations in tumors from vehicle or ADT-030 treatment. N. macrophages, O. F4/80+ PD-L1+ macrophages, P. M1 macrophages, Q. M2 macrophages, R. M1/M2 ratio in KPC tumors after vehicle or ADT-030 treatment. S. Percentage of total dendritic cells/mg tumor, percentage of T. cDC1, and U. cDC2/mg tumor after ADT-030 vs. vehicle treatment. All quantitative data represents the meanL±LSEM. Welch t-test was used for statistical analysis. ns, non-significant, ∗pL<L0.05, ∗∗pL<L0.01, ∗∗∗pL<L0.001, and ∗∗∗∗pL<L0.0001. [file media-11.jpg]

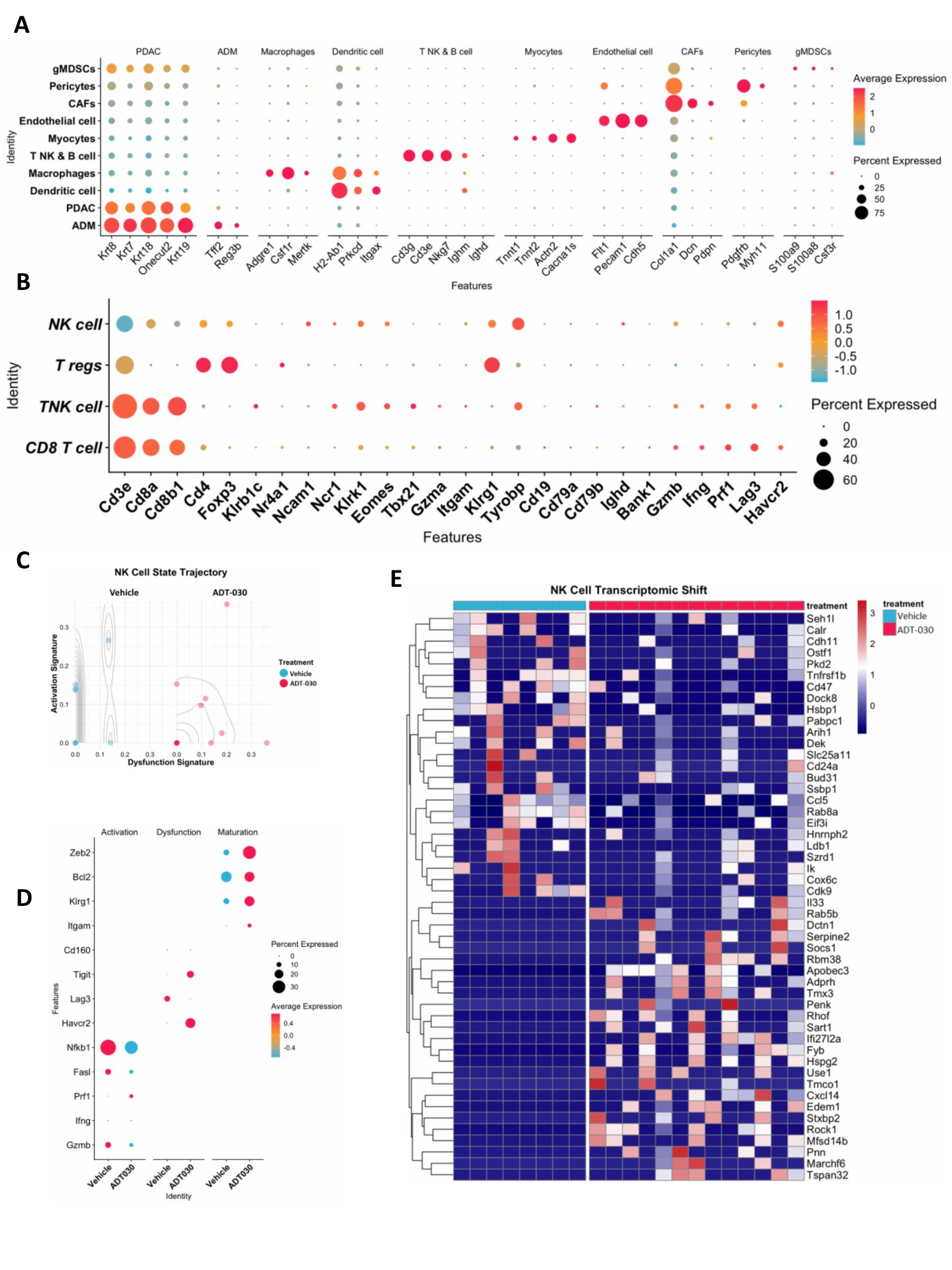

Supplement: Supplement 12 — Supplementary Figure 12. A. Dot plot showing the markers used to identify various clusters such as PDAC, ADM, macrophages, dendritic cells, T NK & B cells, myocytes, endothelial cells, CAFs, pericytes, and gMDCs. B. Dot plot showing the markers used to identify NK cells, T regs, TNK cells, and CD8 T cells. C. NK global state trajectory plot displaying pan-activation vs. pan-dysfunction signatures in vehicle and ADT-030 treated tumors. D. Dot plot showing expression of activation, dysfunction, and maturation genes in NK cells from vehicle and ADT-030 treated tumors with dot size representing the percentage of expressing cells. E. Heat map of differentially expressed genes in NK cells representing a global transcriptional shift toward an activated, cytotoxic program in ADT-030-treated tumors compared to vehicle. [file media-12.jpg]

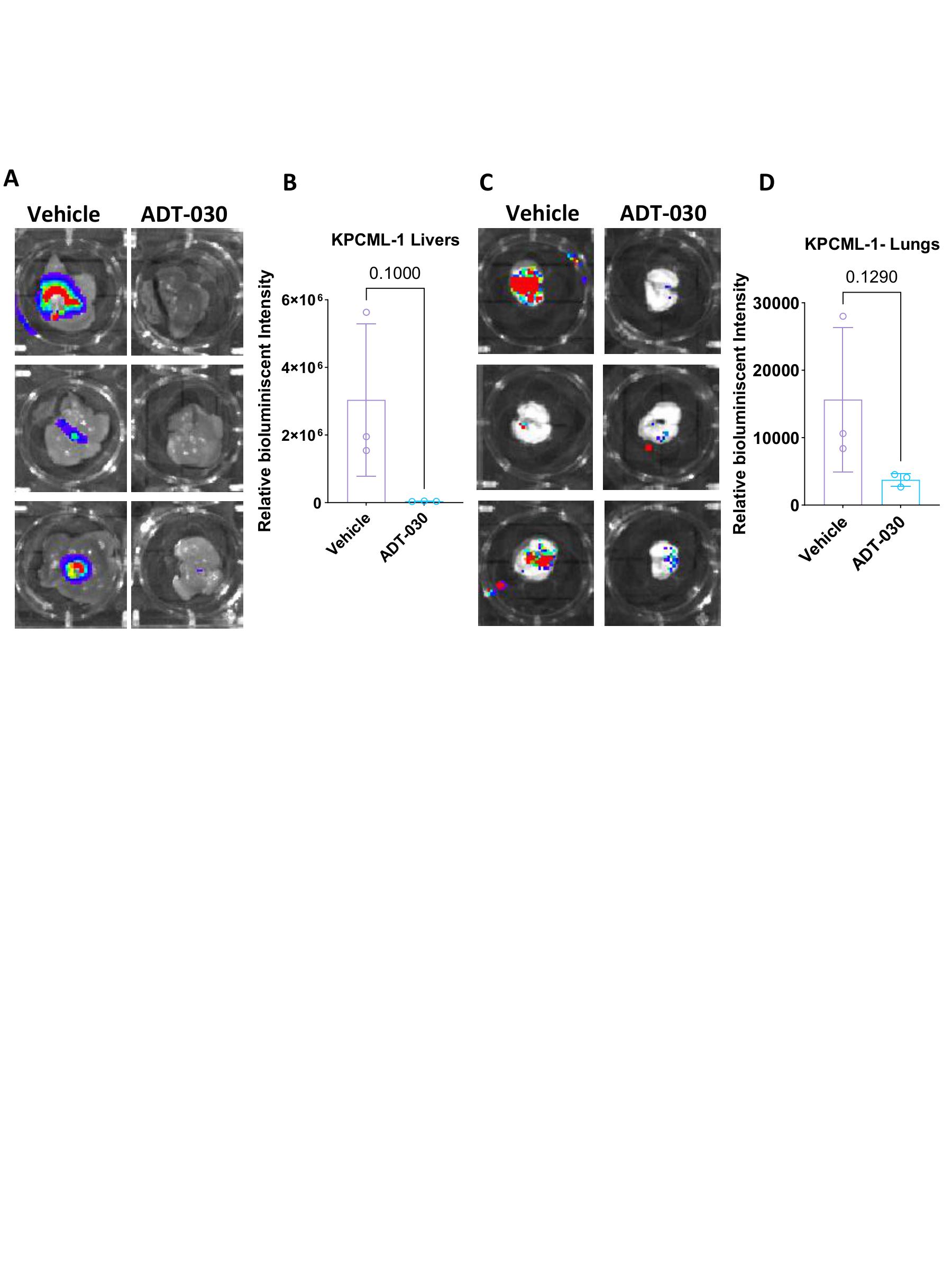

Supplement: Supplement 13 — Supplementary Figure 13: A-B. Ex vivo imaging of livers of KPCML-1-implanted mice treated with vehicle or ADT-030 (A) and bar graph representing the bioluminescence quantification (B). C-D. Ex vivo imaging of lungs in vehicle or ADT-030-treated mice showing reduced metastasis (C) and bar graph representing number of distant metastases (D). Welch t-test was used for statistical analysis. pLvalues are listed on bar graphs. [file media-13.jpg]

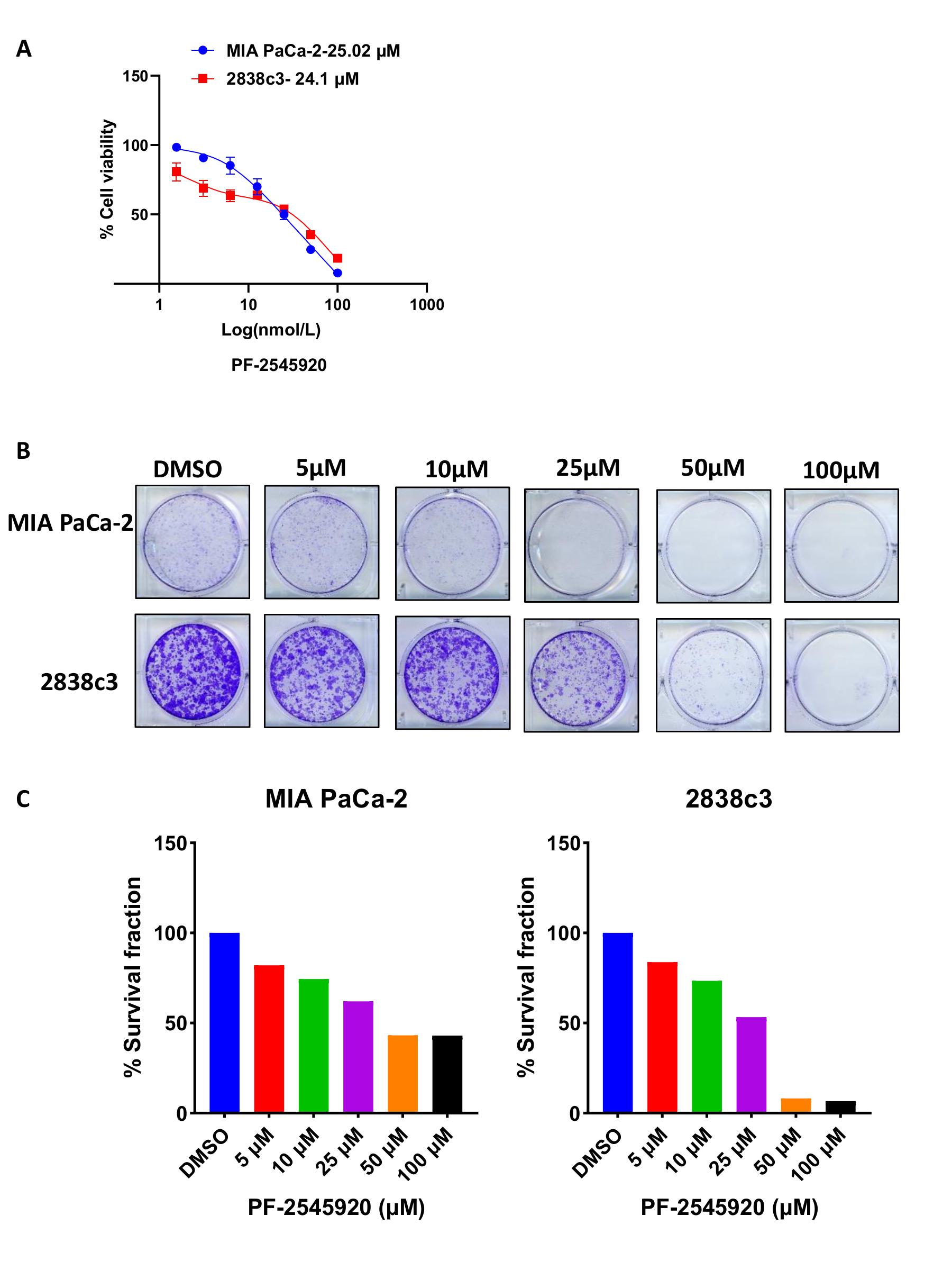

Supplement: Supplement 14 — Supplementary Figure 14. A. Indicated PDAC cell lines were treated with various concentrations of PF-25465920 for three days followed by determining viable cell number using MTT assays. Relative percentage cell viability was plotted with respect to vehicle (DMSO) treated cells. B-C. The indicated PDAC cell lines were treated with various concentrations of PF-2545920 for 2–4 weeks, and long-term cell survival was measured using clonogenic assays. Representative images are shown in (B) and quantification plotted in (C). [file media-14.jpg]

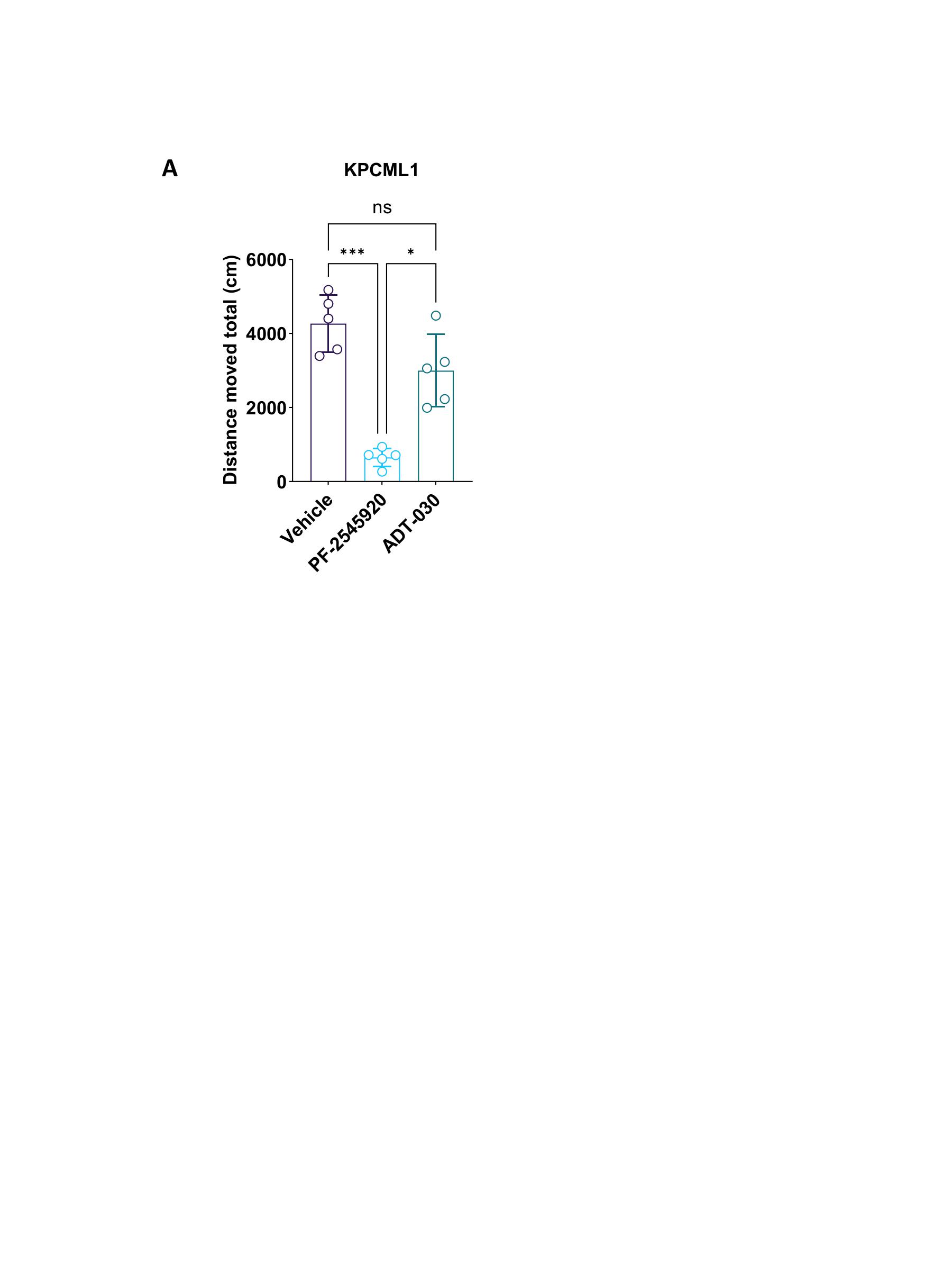

Supplement: Supplement 15 — Supplementary Figure 15. A. Open field and locomotor assay revealed that mice treated with ADT-030 did not show differences in their mobility compared to vehicle while PF-2545920 produced significant reduction in mobility revealing CNS toxicity. ns, non-significant, ∗pL<L0.05, and ∗∗∗pL<L0.001. [file media-15.jpg]

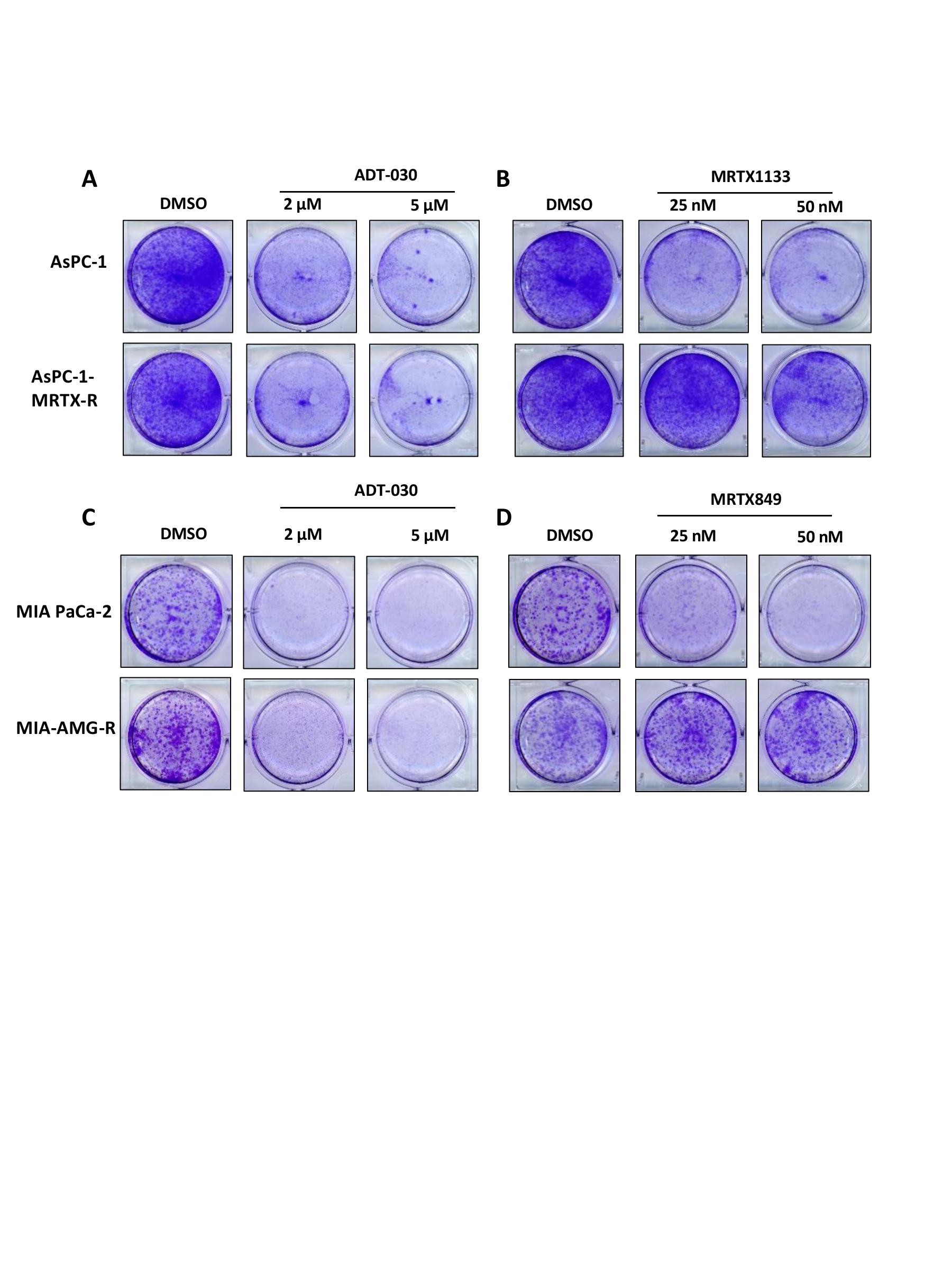

Supplement: Supplement 16 — Supplementary Figure 16. The indicated PDAC cell lines were treated with various concentrations of ADT-030 (A & C), MRTX1133 (B), or MRTX849 (D) for 2–4 weeks, and long-term cell survival was measured using clonogenic assays. [file media-16.jpg]

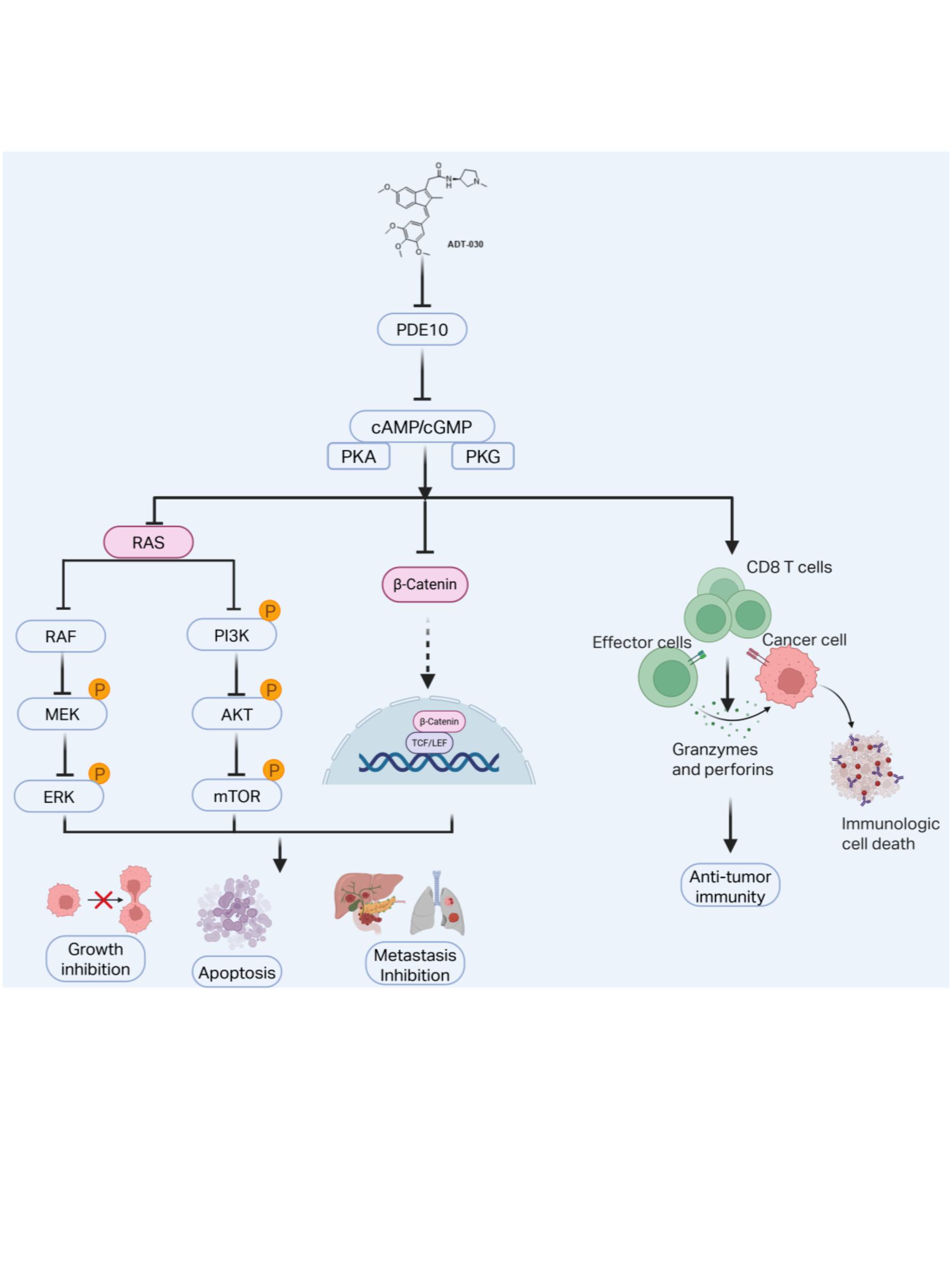

Supplement: Supplement 17 — Supplementary Figure 17. Schematic illustration of the proposed mechanism of action of ADT-030. Inhibition of PDE10 by ADT-030 leads to accumulation of cAMP/cGMP and concomitant activation of PKA/PKG, resulting in both direct antitumor activity and stimulation of antitumor immunity. Direct effects on growth inhibition, induction of apoptosis, and inhibition of metastasis are mediated by suppression of β-catenin/TCF-LEF transcriptional activity and inhibition of both ERK1/2 and PI3K signaling downstream of RAS. Anti-tumor immune effects of ADT-030 are characterized by CD8 T cell-mediated cytotoxicity and immunologic cell death. [file media-17.jpg]
