# Supplementary material for: ADT-030, a novel PDE10 inhibitor, demonstrates potent antitumor activity in pancreatic ductal adenocarcinoma
Source: bioRxiv. 2026 Feb 13:2026.02.11.705411. Preprint. [Version 1] doi: 10.64898/2026.02.11.705411 (PMC12918786; doi:10.64898/2026.02.11.705411)
Supplement: Supplement 18 [file NIHPP2026.02.11.705411v1-supplement-18.pdf]

bioRxiv preprint doi: https://doi.org/10.64898/2026.02.11.705411; this version posted February 14, 2026. The copyright holder for this preprint (which was not certified by peer review) is the author/funder, who has granted bioRxiv a license to display the preprint in perpetuity. It is made available under aCC-BY 4.0 International license.

Marker	Clone	Supplier-Cat #	Dilution
PD-1 FITC	29F.1A12	BioLegend, 135214	1-200
CD206 FITC	C068C2	BioLegend, 141703	1-200
TIM-3 PE	ID4B	BioLegend, 121607	1-1000
F4/80 PE	BM8	BioLegend, 123110	1-800
LAG-3 PECy7	9D3,1C8	BioLegend, 517008	1-200
Ly6C PECy7	HK1.4	BioLegend, 128017	1-3000
CD11b PE CF594	M1/70	BioLegend, 101255	1-3000
CD45 PerCP	30-F11	BioLegend, 103130	1-800
Foxp3 APC	FJK-16s	Invitrogen, 17-5773-82	1-200^{Nuclear}
CD11c APC	N418	BioLegend, 117309	1-400
CTLA4 APC R700	MP6-XT22	BD, 565778	1-1000^{Cytoplasm}
CD172α AF700	P84	BioLegend, 144022	1-1000
CD62L APC Cy7	MEL-14	BioLegend, 104428	1-800
CD86 APC Cy7	GL1	BioLegend, 105045	1-200

bioRxiv preprint doi: https://doi.org/10.64898/2026.02.11.705411; this version posted February 14, 2026. The copyright holder for this preprint (which was not certified by peer review) is the author/funder, who has granted bioRxiv a license to display the preprint in perpetuity. It is made available under aCC-BY 4.0 International license.

CD3 BV 421	145-2C11	BioLegend, 100341	1-800
XCR1 BV 421	ZET	BioLegend, 148216	1-1000
Live/Dead Aqua		Invitrogen, L34966	1-500
TCR γδ BV 605	GL3	BioLegend, 118219	1-800
PD-L1 BV 605	MIH5	BioLegend, 153606	1-400
CD4 BV 650	GK1.5	BioLegend, 100469	1-800
CD103 BV 711	2E7	BioLegend, 121435	1-800
NK1.1 BV 711	PK136	BioLegend, 108475	1-400
CD8 BV 785	53-6.7	BioLegend, 100750	1-800
CCR7 BV 785	4B12	BioLegend, 120217	1-250
CD69 BUV 395	H1.2F3	BD, 569367	1-400
B220 BUV 395	RA3.6B2	BD, 563793	1-400
CD44 BUV 737	IM7	BD, 612799	1-800
CD16/CD32	93	BioLegend, 101320	1-500

Supplementary Table 1. Details on antibodies used for multi-parameter flow cytometry.
